# Supplementary material for: Association between antibiotics and adverse oncological outcomes in patients receiving targeted or immune-based therapy for hepatocellular carcinoma
Source: JHEP Rep. 2023 Mar 30;5(6):100747. doi: 10.1016/j.jhepr.2023.100747 (PMC10183666; doi:10.1016/j.jhepr.2023.100747)
Supplement: Multimedia component 1 [file mmc1.pdf]

Association between antibiotics and adverse oncological outcomes in patients receiving targeted or immune-based therapy for hepatocellular carcinoma

David J. Pinato, Xiaoxue Li, Pallavi Mishra-Kalyani, Antonio D'Alessio, Claudia A. M. Fulgenzi, Bernhard Scheiner, Matthias Pinter, Guo Wei, Julie Schneider, Donna R. Rivera, Richard Pazdur, Marc R. Theoret, Sandra Casak, Steven Lemery, 'Lola Fashoyin-Aje, Alessio Cortellini, Lorraine Pelosof

Table of contents

FIGURES

Fig. S1. **Page 3**
Kaplan-Meier curves depicting the effect of antibiotic use on progression-free survival (Panels A, B) and overall survival (Panels C, D) before (A, C) and after (B, D) propensity score weighting.

TABLES

Table S1 **Page 6**
Use of antibiotics within 30 days of initiation of study treatment

Table S2 **Page 7**
Baseline patient characteristics stratified by antibiotic exposure prior to and after inverse probability of treatment weighting (IPTW): Tyrosine kinase inhibitor group.

Table S3 **Page 8**
Baseline patient characteristics stratified by antibiotic exposure prior to and after inverse probability of treatment weighting (IPTW): immunotherapy group.

Table S4 **Page 9**

Baseline patient characteristics stratified by antibiotic exposure prior to and after inverse probability of treatment weighting (IPTW): placebo group.

Table S5 **Page 10**

The relationship between antibiotic use and efficacy outcomes in patients with unresectable/advanced HCC treated with immune checkpoint inhibitor (ICI) combinations or monotherapy.

Table S6 **Page 11**

Baseline patient characteristics stratified by antibiotic exposure prior to and after propensity score matching: Tyrosine kinase inhibitor group.

Table S7 **Page 12**

Baseline patient characteristics stratified by antibiotic exposure prior to and after propensity score matching: immunotherapy group.

Table S8 **Page 13**

Baseline patient characteristics stratified by antibiotic exposure prior to and after propensity score matching: placebo group.

Table S9 **Page 14**

The relationship between antibiotic use and efficacy outcomes in patients with unresectable/advanced HCC before and after propensity score matching.

Table S10 **Page 15**

The relationship between antibiotic use and efficacy outcomes in patients with unresectable/advanced HCC treated with immune checkpoint inhibitor (ICI) combinations or monotherapy before and after propensity score matching.

Figures

Fig. S1. Kaplan-Meier curves depicting the effect of antibiotic use on PFS (Panels A, B) and OS (Panels C, D) before (A, C) and after (B, D) IPTW.

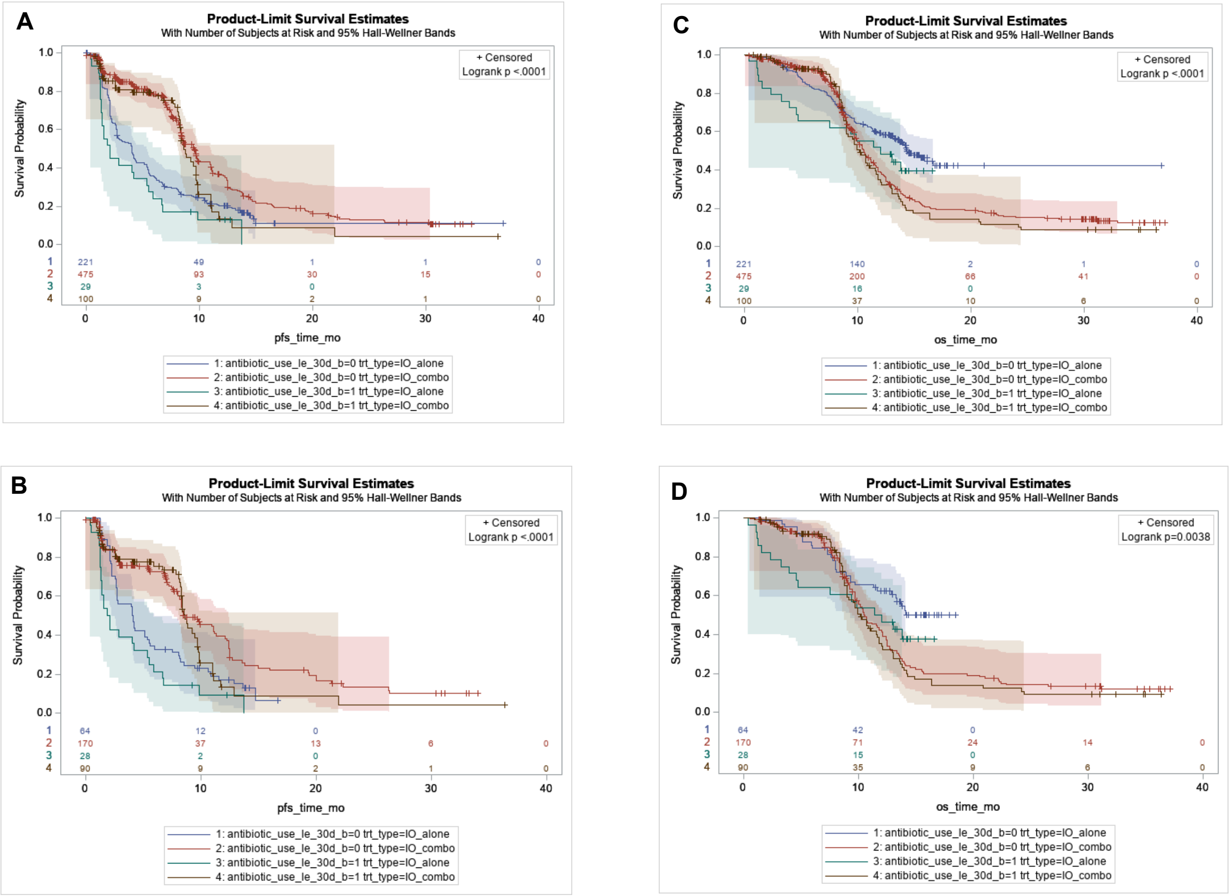

TABLES

Table S1. Use of antibiotics within 30 days of initiation of study treatment.

**Antibiotic Use**	**Placebo (n = 808)**	**Tyrosine kinase inhibitor (n = 1968)**	**Vascular Endothelial Growth Factor (VEGF) inhibitor (n = 480)**	**Immunotherapy (n = 842)**	**All patients (n = 4098)**
None or not within 30 days	708 (88%)	1656 (84%)	407 (85%)	707 (84%)	3478 (85%)
Used within 30 days	100 (12%)	312 (16%)	73 (15%)	135 (16%)	620 (15%)
**Antibiotic Duration**					
≤10 days of exposure	49 (49%)	140 (45%)	41 (56%)	51 (38%)	281 (45%)
>10 days of exposure	51 (51%)	172 (55%)	32 (44%)	84 (62%)	339 (55%)

Table S2. Baseline patient characteristics stratified by antibiotic exposure prior to and after inverse probability of treatment weighting (IPTW): TKI group.

	**No antibiotic use** N = 1640 Effect size = 1640		**Antibiotic use** N = 303 Effect size = 303		**Mean Squared Differences before Propensity Score Weighting**	**No antibiotic use** N = 1469 Effect size = 1746.74		**Antibiotic use** N = 278 Effect size = 1752.47		**Mean Squared Differences before Propensity Score Weighting**
**Variable**	**Mean/N**	**STD/%**	**Mean/N**	**STD/%**		**Mean/N**	**STD/%**	**Mean/N**	**STD/%**	
**Age**										
Mean/SD	61.96	11.67	62.38	11.70	0.035	61.87	12.53	61.91	29.09	0.002
Median/Range	63	19-88	63	27-88		63	19-88	62	27-88	
**Race**										
Asian	899	54.8	166	54.8	-0.001	977	55.9	975	55.6	-0.006
Other/Not reported	131	8.0	33	10.9	0.10	162	9.3	156	8.9	-0.01
White	610	37.2	104	34.3	-0.06	607	34.8	622	35.5	0.01
**Sex**										
Female	247	15.1	64	21.1	0.16	274	15.7	273	15.6	-0.003
Male	1393	84.9	239	78.9	-0.16	1473	84.3	1480	84.4	0.003
**Region**										
Asia	836	51.0	153	50.5	-0.01	903	51.7	905	51.7	-0.001
Rest of world	804	49.0	150	49.5	0.01	844	48.3	847	48.3	0.001
**ECOG PS**										
0	1010	61.6	178	58.7	-0.06	1079	61.8	1110	63.3	0.03
1	630	38.4	125	41.3	0.06	667	38.2	642	36.7	-0.03
**Etiology**										
Non-viral	353	21.5	68	22.4	0.02	420	24.0	427	24.3	0.01
Viral	1118	68.2	210	69.3	0.02	1327	76.0	1326	75.7	-0.01
**Prior Systemic Therapy**										
Yes	957	58.4	154	50.8	-0.15	917	52.5	924	52.7	0.004
No	683	41.7	149	49.2	0.15	830	47.5	828	47.3	-0.004
**Macrovascular**										
Absence	1224	74.6	218	71.9	-0.06	1289	73.8	1298	74.1	0.006
Presence	414	25.2	85	28.1	0.06	458	26.2	455	25.9	-0.006
**Extrahepatic**										
Absence	572	34.9	88	29.0	-0.13	597	34.2	606	34.6	0.01
Presence	1068	65.1	215	71.0	0.13	1150	65.8	1147	65.4	-0.01

Table S3. Baseline patient characteristics stratified by antibiotic exposure prior to and after inverse probability of treatment weighting (IPTW): immunotherapy group.

	**No antibiotic use** N = 696 Effect size = 696		**Antibiotic use** N = 129 Effect size = 129		**Mean Squared Differences before Propensity Score Weighting**	**No antibiotic use** N = 660 Effect size = 779.56		**Antibiotic use** N = 120 Effect size = 788.67		**Mean Squared Differences before Propensity Score Weighting**
**Variable**	**Mean/N**	**STD/%**	**Mean/N**	**STD/%**		**Mean/N**	**STD/%**	**Mean/N**	**STD/%**	
**Age**										
Mean/SD	62.75	11.51	63.39	11.80	0.05	63.49	11.70	62.99	11.76	-0.04
Median/Range	64	18-88	64	34-88		64	18-88	64	34-88	
**Race**										
Asian	337	48.4	65	50.4	0.04	397	50.9	408	51.8	0.02
Other/Not reported	35	5.0	11	8.5	0.14	45	5.7	42	5.3	-0.02
White	312	44.8	51	39.5	-0.11	338	43.3	338	42.9	-0.01
**Sex**										
Female	129	18.5	28	21.7	0.08	145	18.5	148	18.8	0.005
Male	567	81.5	101	78.3	-0.08	635	81.5	641	81.2	-0.005
**Region**										
Asia	307	44.1	53	41.1	-0.06	356	45.7	368	46.6	0.02
Rest of world	388	55.7	76	58.9	0.06	423	54.3	421	53.4	-0.02
**ECOG PS**										
0	434	62.4	72	55.8	-0.13	473	60.6	472	59.9	-0.015
1	262	37.6	57	44.2	0.13	307	39.4	316	40.1	0.015
**Etiology**										
Non-viral	222	31.9	49	38.0	0.13	255	32.7	252	32.0	-0.01
Viral	458	65.8	74	57.4	-0.17	525	67.3	537	68.0	0.01
**Prior Systemic Therapy**										
No	355	51.0	76	58.9	0.16	395	50.7	390	49.4	-0.03
Yes	341	49.0	53	41.1	-0.16	384	49.3	399	50.6	0.03
**Immunotherapy**										
Monotherapy	221	31.8	29	22.5	-0.21	240	30.9	263	33.3	0.05
Combination	475	68.2	100	77.5	0.21	539	69.1	526	66.7	-0.05
**Macrovascular**										
Absence	478	68.7	90	69.8	0.02	533	68.3	552	69.9	0.03
Presence	218	31.3	38	29.5	-0.04	247	31.7	237	30.1	-0.03
**Extrahepatic**										
Absence	236	33.9	38	29.5	-0.10	249	31.9	276	35.0	0.07
Presence	460	66.1	91	70.5	0.10	531	68.1	513	65.0	-0.07

Table S4. Baseline patient characteristics stratified by antibiotic exposure prior to and after inverse probability of treatment weighting (IPTW): placebo group.

Variable	No antibiotic use N = 697 Effect size = 697		Antibiotic use N = 97 Effect size = 97		Mean Squared Differences before Propensity Score Weighting	No antibiotic use N = 675 Effect size = 766.69		Antibiotic use N = 92 Effect size = 722.45		Mean Squared Differences before Propensity Score Weighting
	**Mean/N**	**STD/%**	**Mean/N**	**STD/%**		**Mean/N**	**STD/%**	**Mean/N**	**STD/%**	
**Age**										
Mean/STD	62.50	11.09	62.55	12.11	0.004	62.26	11.61	62.01	11.62	-0.02
Median/Range	63	23-86	63	24-85		63	23-86	63	24-85	
**Race**										
Asian	300	43.0	38	39.2	-0.08	335	43.7	339	43.9	0.005
Other/Not reported	69	9.9	9	9.3	-0.02	78	10.2	85	11.0	0.03
White	315	45.2	46	47.4	0.04	354	46.1	348	45.1	-0.02
**Sex**										
Female	89	12.8	24	24.7	0.31	106	13.8	104	13.4	-0.01
Male	608	87.2	73	75.3	-0.31	661	86.2	669	86.6	0.01
**Region**										
Asia	432	62.0	61	62.9	-0.02	298	38.9	309	40.0	0.02
Rest of world	418	60.0	45	46.4	0.02	469	61.1	463	60.0	-0.02
**ECOG PS**										
0	279	40.0	52	53.6	-0.27	452	59.0	458	59.2	0.01
1	10	1.4	1	1.0	0.27	314	41.0	315	40.8	-0.01
**Etiology**										
Non-viral	264	37.9	30	30.9	-0.15	286	37.3	304	39.3	0.04
Viral	423	60.7	66	68.0	0.15	480	62.7	469	60.7	-0.04
**Prior Systemic Therapy**										
No	0	-	0	-	-	0	-	0	-	-
Yes	697	-	97	-	-	766.69	-	772.45	-	-
**Macrovascular**										
Absence	489	70.2	62	63.9	-0.13	536	69.9	550	71.3	0.03
Presence	208	29.8	35	36.1	0.13	231	30.1	222	28.7	-0.03
**Extrahepatic**										
Absence	197	28.3	26	26.8	-0.03	214	27.9	208	26.9	-0.02
Presence	500	71.7	71	73.2	0.03	553	72.1	565	73.1	0.02

Table S5. The relationship between antibiotic use and efficacy outcomes in patients with unresectable/advanced HCC treated with immune checkpoint inhibitor (ICI) combinations or monotherapy.

	**Unadjusted Analyses**		**Adjusted using IPTW ***	
	**No Antibiotic Use**	**Antibiotic Use**	**No Antibiotic Use**	**Antibiotic Use**
**Immune checkpoint inhibitor combinations**				
Sample size				
# patients	475	100	448	92
Effect size	475	100	539.76	542.65
**Progression-free survival (PFS)**				
Median (95% CI), mo	9.6 (8.5, 9.8)	8.6 (8.3, 9.7)	9.7 (8.5, 11.0)	8.6 (8.3, 9.7)
HR** (95% CI)	1.33 (0.97, 1.81)		1.50 (1.26, 1.77)	
**Overall survival (OS)**				
Median (95% CI), mo	10.5 (10.0, 11.2)	10.2 (9.0, 11.6)	10.6 (10.2, 11.4)	9.9 (9.0, 11.6)
HR** (95% CI)	1.13 (0.87, 1.47)		1.14 (0.99, 1.32)	
**Immune checkpoint inhibitor monotherapy**				
Sample size				
# patients	221	29	212	28
Effect Size	221	29	240.20	228.58
**Progression-free survival (PFS)**				
Median (95% CI), mo	4.0 (2.8, 4.4)	2.1 (1.3, 5.4)	4.0 (2.8, 4.4)	2.1 (1.3, 5.4)
HR (95% CI)	1.51 (1.00, 2.28)		1.49 (1.22 ,1.80)	
**Overall survival (OS)**				
Median (95% CI), mo	14.3 (13.2, NE)	12.0 (4.6, NE)	14.4 (13.2, NE)	13.0 (4.6, NE)
HR (95% CI)	1.47 (0.88, 2.45)		1.31 (1.02, 1.68)	

** The weights were derived from a propensity score (IPTW) models which included the following baseline variables as covariates: age, race, gender, region, European Collaborative Oncology Group (ECOG) performance status, etiology of chronic liver disease, presence of macrovascular invasion, presence of extrahepatic disease and receipt of prior lines of treatment.*

*** HR is stratified by immune checkpoint inhibitor alone or immune checkpoint inhibitor combination.*

Table S6. Baseline patient characteristics stratified by antibiotic exposure prior to and after propensity score matching: Tyrosine kinase inhibitor (TKI) group.

Variable	No antibiotic use N = 1640		Antibiotic use N = 303		Mean Squared Differences before matching	No antibiotic use N = 548		Antibiotic use N = 276		Mean Squared Differences after matching
	Mean/N	STD/%	Mean/N	STD/%		Mean/N	STD/%	Mean/N	STD/%	
**Age**	61.96	11.67	62.38	11.70	0.035	62.26	11.61	62.01	11.62	-0.021
**Race**										
Asian	899	54.8	166	54.8	-0.001	296	54.0	149	54.0	-0.001
Other/Not reported	131	8.0	33	10.9	0.10	64	11.7	33	12.0	0.01
White	610	37.2	104	34.3	-0.06	188	34.3	94	34.1	-0.01
**Sex**										
Female	247	15.1	64	21.1	0.16	107	19.5	57	20.7	0.03
Male	1393	84.9	239	78.9	-0.16	441	80.5	219	79.3	-0.03
**Region**										
Asia	836	51.0	153	50.5	-0.01	271	49.5	138	50.0	0.01
Rest of world	804	49.0	150	49.5	0.01	277	50.5	138	50.0	-0.01
**ECOG**										
0	1010	61.6	178	58.7	-0.06	325	59.3	161	58.3	-0.02
1	630	38.4	125	41.3	0.06	223	40.7	115	41.7	0.02
**Etiology**										
Non-viral	353	21.5	68	22.4	0.02	135	24.6	68	24.6	0.00
Viral	1118	68.2	210	69.3	0.02	413	75.4	208	75.4	0.00
**Prior Therapy**										
Yes	957	58.4	154	50.8	-0.15	265	48.4	129	46.7	-0.03
No	683	41.7	149	49.2	0.15	283	51.6	147	53.3	0.03
**Macrovascular**										
Absence	1224	74.6	218	71.9	-0.06	396	72.3	200	72.5	0.004
Presence	414	25.2	85	28.1	0.06	152	27.7	76	27.5	-0.004
**Extrahepatic**										
Absence	572	34.9	88	29.0	-0.13	149	27.2	79	28.6	0.03
Presence	1068	65.1	215	71.0	0.13	399	72.8	197	71.4	-0.03

Table S7. Baseline patient characteristics stratified by antibiotic exposure prior to and after propensity score matching: immunotherapy group.

Variable	No antibiotic use N = 696		Antibiotic use N = 129		Mean Squared Differences before matching	No antibiotic use N = 234		Antibiotic use N = 118		Mean Squared Differences after matching
	Mean/N	STD/%	Mean/N	STD/%		Mean/N	STD/%	Mean/N	STD/%	
**Age**	62.75	11.51	63.39	11.80	0.05	63.49	11.70	62.99	11.76	-0.04
**Race**										
Asian	337	48.4	65	50.4	0.04	115	49.1	62	52.5	0.07
Other/Not reported	35	5.0	11	8.5	0.14	21	9.0	10	8.5	-0.02
White	312	44.8	51	39.5	-0.11	98	41.9	46	39.0	-0.06
**Sex**										
Female	129	18.5	28	21.7	0.08	47	20.1	24	20.3	0.006
Male	567	81.5	101	78.3	-0.08	187	79.9	94	79.7	-0.006
**Region**										
Asia	307	44.1	53	41.1	-0.06	95	40.6	51	43.2	0.05
Rest of world	388	55.7	76	58.9	0.06	139	59.4	67	56.8	-0.05
**ECOG**										
0	434	62.4	72	55.8	-0.13	133	56.8	67	56.8	-0.001
1	262	37.6	57	44.2	0.13	101	43.2	51	43.2	0.001
**Etiology**										
Non-viral	222	31.9	49	38.0	0.13	96	41.0	46	39.0	-0.04
Viral	458	65.8	74	57.4	-0.17	138	59.0	72	61.0	0.04
**Prior Therapy**										
No	355	51.0	76	58.9	0.16	122	52.1	66	55.9	0.08
Yes	341	49.0	53	41.1	-0.16	112	47.9	52	44.1	-0.08
**Immunotherapy**										
Monotherapy	221	31.8	29	22.5	-0.21	64	27.4	28	23.7	-0.08
Combination	475	68.2	100	77.5	0.21	170	72.6	90	76.3	0.08
**Macrovascular**										
Absence	478	68.7	90	69.8	0.02	158	67.5	81	68.6	0.02
Presence	218	31.3	38	29.5	-0.04	76	32.5	37	31.4	-0.02
**Extrahepatic**										
Absence	236	33.9	38	29.5	-0.10	64	27.4	34	28.8	0.03
Presence	460	66.1	91	70.5	0.10	170	72.6	84	71.2	-0.03

Table S8. Baseline patient characteristics stratified by antibiotic exposure prior to and after propensity score matching: placebo group.

Variable	No antibiotic use N = 697		Antibiotic use N = 97		Mean Squared Differences before matching	No antibiotic use N = 175		Antibiotic use N = 91		Mean Squared Differences after matching
	Mean/N	STD/%	Mean/N	STD/%		Mean/N	STD/%	Mean/N	STD/%	
**Age**	62.50	11.09	62.55	12.11	0.004	62.77	10.72	62.56	12.14	-0.018
**Race**										
Asian	300	43.0	38	39.2	-0.08	69	39.4	37	40.7	0.025
Other/Not reported	69	9.9	9	9.3	-0.02	21	12.0	9	9.9	-0.07
White	315	45.2	46	47.4	0.04	85	48.6	45	49.5	0.02
**Sex**										
Female	89	12.8	24	24.7	0.31	41	23.4	23	25.3	0.04
Male	608	87.2	73	75.3	-0.31	134	76.6	68	74.7	-0.04
**Region**										
Asia	432	62.0	61	62.9	-0.02	63	36.0	35	38.5	0.05
Rest of world	418	60.0	45	46.4	0.02	112	64.0	56	61.5	-0.05
**ECOG**										
0	279	40.0	52	53.6	-0.27	90	51.4	44	48.4	-0.06
1	10	1.4	1	1.0	0.27	85	48.6	47	51.6	0.06
**Etiology**										
Non-viral	264	37.9	30	30.9	-0.15	50	28.6	28	30.8	0.05
Viral	423	60.7	66	68.0	0.15	125	71.4	63	69.2	-0.05
**Prior Therapy**										
No	0	-	0	-	-	0	-	0	-	-
Yes	697	-	97	-	-	175	-	91	-	-
**Macrovascular**										
Absence	489	70.2	62	63.9	-0.13	122	69.7	59	64.8	-0.10
Presence	208	29.8	35	36.1	0.13	53	30.3	32	35.2	0.10
**Extrahepatic**										
Absence	197	28.3	26	26.8	-0.03	54	30.9	23	25.3	-0.12
Presence	500	71.7	71	73.2	0.03	121	69.1	68	74.7	0.12

Table 9. The relationship between antibiotic use and efficacy outcomes in patients with unresectable/advanced HCC before and after propensity score matching.

	Unadjusted Analyses		Adjusted using propensity score	
	**No Antibiotic Use**	**Antibiotic Use**	**No Antibiotic Use**	**Antibiotic Use**
**All patients**				
Sample size				
# patients	3435	601	1091	550
**Progression-free survival (PFS)**				
Median (95% CI), months	4.2 (4.1, 4.5)	3.6 (3.0, 4.0)	5.4 (4.3, 5.5)	3.6 (3.0, 4.0)
HR* (95% CI)	1.36 (1.23, 1.50)		1.39 (1.23 ,1.57)	
**Overall survival (OS)**				
Median (95% CI), months	10.8 (10.4, 11.3)	8.6 (7.9, 9.1)	11.1 (10.4, 11.8)	8.6 (7.8, 9.2)
HR* (95% CI)	1.42 (1.29, 1.57)		1.41 (1.25 ,1.59)	
**Tyrosine kinase inhibitor Group**				
Sample size				
# patients	1640	303	548	276
**Progression-free survival (PFS)**				
Median (95% CI), months	5.6 (5.5, 5.7)	3.7 (3.6, 4.3)	5.5 (4.7 ,5.6)	3.7 (3.6 ,4.3)
HR (95% CI)	1.40 (1.22, 1.61)		1.27 (1.08 ,1.51)	
**Overall survival (OS)**				
Median (95% CI), months	12.2 (11.6, 13.1)	8.4 (7.1, 9.8)	12.1 (10.7 ,13.9)	8.6 (7.1 ,10.1)
HR (95% CI)	1.51 (1.31, 1.73)		1.41 (1.19 ,1.68)	
**Immunotherapy Group**				
Sample size				
# patients	696	129	234	118
**Progression-free survival (PFS)**				
Median (95% CI), months	8.2 (7.6, 8.4)	8.3 (6.8, 8.9)	8.1 (7.0, 8.5)	8.3 (6.7, 8.6)
HR* (95% CI)	1.39 (1.08, 1.78)		1.41 (1.06, 1.71)	
**Overall survival (OS)**				
Median (95% CI), months	11.4 (10.6, 12.1)	10.7 (9.1, 11.8)	11.8 (10.4 ,12.7)	10.7 (9.1 ,11.8)
HR* (95% CI)	1.19 (0.94 ,1.50)		1.19 (0.91 ,1.57)	
**Placebo Group**				
Sample size				
# patients	697	97	175	91
**Progression-free survival (PFS)**				
Median (95% CI), months	1.87 (1.81, 1.93)	1.61 (1.41, 1.84)	1.8 (1.7 ,2.1)	1.61 (1.41 ,1.91)
HR (95% CI)	1.29 (1.03, 1.62)		1.31 (1.00 ,1.71)	
**Overall survival (OS)**				
Median (95% CI), months	8.2 (7.4, 8.9)	4.4 (3.7, 6.6)	6.87 (5.45 ,9.40)	4.17 (3.45 ,5.88)
HR (95% CI)	1.58 (1.25, 1.99)		1.54 (1.16 ,2.05)	

** HR is stratified by treatment group (tyrosine kinase inhibitor or immune checkpoint inhibitor alone or immune checkpoint inhibitor combination or Placebo or VEGF for all patients, immune checkpoint inhibitor alone or immune checkpoint inhibitor combination for immune checkpoint inhibitor group).*

Table S10. The relationship between antibiotic use and efficacy outcomes in patients with unresectable/advanced HCC treated with immune checkpoint inhibitor (ICI) combinations or monotherapy before and after propensity score matching.

	**Unadjusted Analyses**		**Adjusted using propensity score**	
	**No Antibiotic Use**	**Antibiotic Use**	**No Antibiotic Use**	**Antibiotic Use**
**Immune checkpoint inhibitor combinations**				
Sample size				
# patients	475	100	177	90
**Progression-free survival (PFS)**				
Median (95% CI), months	9.6 (8.5, 9.8)	8.6 (8.3, 9.7)	11.0 (8.4, 12.1)	8.6 (8.3, 9.7)
HR (95% CI)	1.33 (0.97, 1.81)		1.61 (1.12, 2.33)	
**Overall survival (OS)**				
Median (95% CI), months	10.5 (10.0, 11.2)	10.2 (9.0, 11.6)	11.4 (10.4, 12.1)	10.2 (9.0, 11.6)
HR (95% CI)	1.13 (0.87, 1.47)		1.23 (0.91, 1.68)	
**Immune checkpoint inhibitor monotherapy**				
Sample size				
# patients	221	29	56	28
**Progression-free survival (PFS)**				
Median (95% CI), months	4.0 (2.8, 4.4)	2.1 (1.3, 5.4)	4.0 (2.6, 5.4)	2.1 (1.3, 4.2)
HR (95% CI)	1.51 (1.00, 2.28)		1.48 (0.92, 2.38)	
**Overall survival (OS)**				
Median (95% CI), months	14.3 (13.2, NE)	12.0 (4.6, NE)	14.4 (10.5, NE)	11.9 (4.6, NE)
HR (95% CI)	1.47 (0.88, 2.45)		1.61 (0.87, 2.97)	

NE = not estimable
