# Supplementary material for: Association between antibiotics and adverse oncological outcomes in patients receiving targeted or immune-based therapy for hepatocellular carcinoma
Source: JHEP Rep. 2023 Mar 30;5(6):100747. doi: 10.1016/j.jhepr.2023.100747 (PMC10183666; doi:10.1016/j.jhepr.2023.100747)
Supplement: Multimedia component 4 [file mmc4.pdf]

Research article

JHEP|Reports

Association between antibiotics and adverse oncological outcomes in patients receiving targeted or immune-based therapy for hepatocellular carcinoma

David J. Pinato,[1,2,*] Xiaoxue Li,[3,†] Pallavi Mishra-Kalyani,[3,†] Antonio D'Alessio,[1,4] Claudia A.M. Fulgenzi,[1,5] Bernhard Scheiner,[1,6] Matthias Pinter,[6] Guo Wei,[7,†] Julie Schneider,[7,†] Donna R. Rivera,[7,†] Richard Pazdur,[7,8,†] Marc R. Theoret,[7,8,†] Sandra Casak,[8,†] Steven Lemery,[8,†] Lola Fashoyin-Aje,[8,†] Alessio Cortellini,[1] Lorraine Pelosof[8,*,†]

[1]*Department of Surgery & Cancer, Imperial College London, Hammersmith Hospital, Du Cane Road, London, UK;* [2]*Division of Oncology, Department of Translational Medicine, University of Piemonte Orientale, Novara, Italy;* [3]*Office of Biostatistics, Center for Drug Evaluation and Research (CDER), US FDA, Silver Spring, MD, USA;* [4]*Department of Biomedical Sciences, Humanitas University, Milan, Italy;* [5]*Department of Medical Oncology, University Campus Bio-Medico of Rome, Rome, Italy;* [6]*Division of Gastroenterology and Hepatology, Department of Internal Medicine III, Medical University of Vienna, Vienna, Austria;* [7]*Oncology Center of Excellence, US FDA, Silver Spring, MD, USA;* [8]*Office of Oncologic Diseases, Center for Drug Evaluation and Research (CDER), US FDA, Silver Spring, MD, USA*

JHEP Reports **2023.** https://doi.org/10.1016/j.jhepr.2023.100747

Background & Aims: Immune checkpoint inhibitors (ICIs) alone or in combination with other ICIs or vascular endothelial growth factor pathway inhibitors are therapeutic options in unresectable/metastatic hepatocellular carcinoma (HCC). Whether antibiotic (ATB) exposure affects outcome remains unclear.

Methods: This study retrospectively analysed an FDA database including 4,098 patients receiving ICI (n = 842) either as monotherapy (n = 258) or in combination (n = 584), tyrosine kinase inhibitor (TKI) (n = 1,968), vascular endothelial growth factor pathway inhibitors (n = 480), or placebo (n = 808) as part of nine international clinical trials. Exposure to ATB within 30 days before or after treatment initiation was correlated with overall survival (OS) and progression-free survival (PFS) across therapeutic modality before and after inverse probability of treatment weighting (IPTW).

Results: Of 4,098 patients with unresectable/metastatic HCC, of which 39% were of hepatitis B aetiology and 21% were of hepatitis C aetiology, 83% were males with a median age of 64 years (range 18–88), a European Collaborative Oncology Group performance status of 0 (60%), and Child–Pugh A class (98%). Overall, ATB exposure (n = 620, 15%) was associated with shorter median PFS (3.6 months in ATB-exposed *vs.* 4.2 months; hazard ratio [HR] 1.29; 95% CI 1.22, 1.36) and OS (8.7 months in ATB-exposed *vs.* 10.6 months; HR 1.36; 95% CI 1.29, 1.43). In IPTW analyses, ATB was associated with shorter PFS in patients treated with ICI (HR 1.52; 95% CI 1.34, 1.73), TKI (HR 1.29; 95% CI 1.19, 1.39), and placebo (HR 1.23; 95% CI 1.11, 1.37). Similar results were observed in IPTW analyses of OS in patients treated with ICI (HR 1.22; 95% CI 1.08, 1.38), TKI (HR 1.40; 95% CI 1.30, 1.52), and placebo (HR 1.40; 95% CI 1.25, 1.57).

Conclusions: Unlike other malignancies where the detrimental effect of ATB may be more prominent in ICI recipients, ATB is associated with worse outcomes in this study across different therapies for HCC including placebo. Whether ATB is causally linked to worse outcomes through disruption of the gut–liver axis remains to be demonstrated in translational studies.

Impact and Implications: A growing body of evidence suggests the host microbiome, frequently altered by antibiotic treatment, as an important outcome predictor in the context of immune checkpoint inhibitor therapy. In this study, we analysed the effects of early antibiotic exposure on outcomes in almost 4,100 patients with hepatocellular carcinoma treated within nine multicentre clinical trials. Interestingly, early exposure to antibiotic treatment was associated with worse outcomes not only in patients treated with immune checkpoint inhibitors but also in those treated with tyrosine kinase inhibitors and placebo. This is in contrast to data published in other malignancies, where the detrimental effect of antibiotic treatment may be more prominent in immune checkpoint inhibitor recipients, highlighting the uniqueness of hepatocellular carcinoma given the complex interplay between cirrhosis, cancer, risk of infection, and the pleiotropic effect of molecular therapies for this disease.

Keywords: Antibiotics; Cancer immunotherapy; Targeted therapy; Immune checkpoint inhibitors; Hepatocellular carcinoma; Gut microbiota; Microbial dysbiosis.
Received 21 January 2023; received in revised form 8 March 2023; accepted 8 March 2023; available online 30 March 2023

[†] XL, PMK, GW, JS, DR, RP, MT, SC, SL, LFA, are and LP are employed by the US FDA. The views expressed in this paper reflect the views of the authors and should not be construed to represent FDA's views or policies.

* Corresponding authors. Addresses: Imperial College London Hammersmith Campus, Du Cane Road, W12 0HS, London, UK. Tel.: +44-20-83833720 (D.J. Pinato); Office of Oncologic Diseases, Center for Drug Evaluation and Research (CDER), US Food and Drug Administration (FDA), 10903 New Hampshire Avenue, White Oak Building 22, Silver Spring, MD 20993, USA. Tel.: +1-240-402-6469 (L. Pelosof).
E-mail addresses: david.pinato@imperial.ac.uk (D.J. Pinato), Lorraine.Pelosof@fda.hhs.gov (L. Pelosof).

ELSEVIER

EASL The Home of Hepatology

© 2023 The Author(s). Published by Elsevier B.V. on behalf of European Association for the Study of the Liver (EASL). This is an open access article under the CC BY license (http://creativecommons.org/licenses/by/4.0/).

Introduction

The advent of effective systemic therapy options has reshaped the therapeutic landscape of advanced/unresectable hepatocellular carcinoma (HCC).[1] Sequential use of tyrosine kinase inhibitor (TKI) therapy represented a breakthrough in an oncological diagnosis that had for a long time not benefited from the use of systemic therapy.[2,3] More recently, clinical testing of monoclonal antibodies in the immune checkpoint inhibitor (ICI) class targeting the programmed cell death-1 (PD-1) receptor/ligand (PD-L1) and the cytotoxic T-cell lymphocyte associated antigen-4 (CTLA-4), two key drivers of anticancer immunity,[4] has led to the understanding that a proportion of patients with HCC may respond to T-cell immune reconstitution.[5] However, in the absence of predictive biomarkers capable of identifying patients who benefit from ICIs, demonstrating the clinical utility of monotherapy regimens has been particularly difficult, especially in view of late-stage failures in clinical development.[6,7]

Clinical testing of combinations of PD-1 pathway inhibitors with blockers of the vascular endothelial growth factor (VEGF) pathway, the CTLA-4 pathway, or TKIs have risen to prominence as therapies with the potential to generate radiologically measurable responses in over one-fourth of patients with advanced HCC.[8] These combinations have also demonstrated survival benefit compared with sorafenib as illustrated in the IMbrave150 trial, which evaluated the combination of atezolizumab plus bevacizumab.[9]

As drug development in HCC continues at a rapid pace, considerable interest has been devoted to the investigation of mechanisms of response and resistance to ICI, in an effort to guide personalised therapy.[10] The specific immune-mediated mechanism of action of ICIs has led to the study of the host immune status as a source of prognostic and predictive traits that may aid clinicians in formulating therapeutic decisions in the clinic. In recipients of ICI therapy, characteristics such as pretreatment BMI,[11] the presence of a systemic pro-inflammatory status,[12,13] the emergence of treatment-related adverse events[14] are independently associated with outcomes from immunotherapy.

In addition to host factors, concomitant therapies may have a direct effect on the host immune function and have been studied for their ability to modify the efficacy of ICI or make patients more prone to developing toxicity from ICI therapy.[15,16]

Antibiotics (ATB) are among the class of drugs that exert immunomodulatory effects, for example, through perturbation of the gut microbiota.[17] Use of broad-spectrum ATB can reduce gut bacterial diversity and foster the expansion of *taxa* that may negatively affect response to ICI.[18,19] Data suggest that ATB exposure either before or early during the course of ICI therapy are associated with worse outcomes in recipients of ICI therapy in terms of objective response rate, progression-free survival (PFS), and overall survival (OS).[20–24] In indications other than HCC, it has been reported that the detrimental effect of ATB is most apparent in patients who receive ICI therapy as compared with those who receive cytotoxic chemotherapy[25] or chemo-immunotherapy combinations,[26] suggesting that ATB might exert a preconditioning effect on cancer-specific immune control.[27]

In patients with HCC the role of ATB exposure in influencing outcome from ICI is unclear.

Two previously published multicentre retrospective studies have reported conflicting results.[28,29] However, significant heterogeneity exists among published studies, where the majority of patients were treated with PD-1 monotherapy across different treatment lines and with varying degrees of liver dysfunction, a factor that affects the prognosis of ICI recipients.[30,31]

To further evaluate the strength and direction of the relationship between ATB exposure and outcome from immunotherapy we performed a patient-level analysis of international clinical trials of systemic therapy (ICI and TKI) in advanced/unresectable HCC.

Patients and methods

Study population

An internal US FDA database was used to identify clinical trials submitted to the FDA between 2016 and 2019 to support marketing applications of systemic anticancer therapies for the treatment of patients with unresectable/metastatic HCC. Nine multicentre trials were included and consisted of treatment arms including placebo, TKI, VEGF inhibitors, and PD-1/PD-L1 inhibitors as monotherapy and in combination with CTLA-4 antagonists, VEGF pathway inhibitors, or TKI. The immunotherapy agents studied included anti-PD-1, anti-PD-L1, and anti-CTLA-4 agents. The TKIs and VEGF inhibitors studied were regorafenib, lenvatinib, sorafenib, cabozantinib, ramucirumab, and bevacizumab. The different trials studied patients in the first-line setting and beyond.

Endpoint definition

OS was defined as the time from randomisation (or date of enrolment for patients in single-arm studies) to death or last recorded follow-up. PFS was defined as the time from randomisation (or enrolment for single-arm studies) to progression or death. PFS was determined according to Response Evaluation Criteria in Solid Tumours (RECIST) v1.1 guidelines. For ATB exposure, concomitant medication records were screened to identify administration of any systemic ATB uses up to 30 days before treatment initiation. Patients who had ATB exposure, defined as exposure within 30 days before and 30 days after the initiation of any anticancer treatment, were of interest. Duration of exposure was recorded and categorised into more *vs.* less than 10 days. All clinical study-related procedures and data collection were indicated by the sponsors to have been conducted in accordance with the Declaration of Helsinki and in accordance with Good Clinical Practice.

Analysis methods

Demographic data were summarised using descriptive statistics. Categorical variables were summarised as proportions, and continuous variables were summarised using medians and ranges. For the analyses on OS and PFS, the Kaplan–Meier product limit method and log-rank tests were used to compare patients who had ATB exposure with patients who did not. Cox proportional hazard models were used to estimate the

hazard ratio (HR) of patients who had ATB exposure compared with patients who did not. All PFS and OS analyses were conducted in all patients as well as by treatment type. Unless otherwise specified, the immunotherapy group included patients receiving PD-1/PD-L1 inhibitors as monotherapy and in combination with CTLA-4 antagonists, VEGF pathway inhibitors, or TKI. Because of the inconsistent association between ATB exposure and outcome in ICI recipients in our dataset and evidence in the literature suggesting that ICI combination therapies might be less susceptible to the detrimental effects of ATB, we also evaluated ICI combination regimens separately from ICI monotherapy.

Inverse probability of treatment weighting (IPTW) was used in attempt to achieve a balanced distribution of confounders across exposure groups. The weights were derived from a propensity score IPTW model that included the following baseline variables as covariates: age, race, sex, region, European Collaborative Oncology Group (ECOG) performance status, aetiology of chronic liver disease, presence of macrovascular invasion, presence of extrahepatic disease, and receipt of prior lines of treatment. The mean squared differences (MSDs) were calculated to measure the balance in observed baseline characteristics between groups of ATB exposure. The MSDs before and after IPTW were calculated for each covariate by ATB exposure group; both unadjusted and adjusted (by IPTW) PFS and OS analyses were conducted. To validate our findings, all statistical analyses were repeated after propensity score matching considering the same potential confounders as for IPTW. All statistical analyses were conducted using the SAS software (SAS Institute Inc., Cary, NC, USA).

Results

Patients

Overall, 4,098 patients were pooled from nine selected clinical trials; among them, 1,968 (48%) received TKIs, 842 (21%) received ICI-based regimens, 480 (12%) received anti-VEGF monoclonal antibodies, and 808 (20%) received placebo. When considering antibiotic therapy exposure in the whole population, 620 patients (15%) had received ATB within the time frame of 30 days before and after first anticancer treatment or placebo was initiated, whereas the remaining 3,478 trial patients (85%) had not been exposed to ATB at all or had received them outside the time frame of interest. Rates of ATB exposure were similar across treatment types ranging from 12% of placebo to 16% of TKI and immunotherapy recipients. Duration of ATB treatment was less than or equal to 10 days in 281 of 620 exposed patients (45%) and comparable across types (Table S1).

Table 1. Baseline characteristics of clinical trial participants stratified according to antibiotic use.

	No antibiotic use n = 3,478		Antibiotic use n = 620	
**Variables**	**Median/n**	**Range/%**	**Median/n**	**Range/%**
**Age**	64	18–88	63	24–88
Race				
Missing	57	1.6	10	1.6
Asian	1,732	49.8	316	51.0
Black	59	1.7	14	2.3
White	1,432	41.2	239	38.5
Not reported	166	4.8	30	4.8
Other	32	0.9	11	1.8
Sex				
Female	538	15.5	139	22.4
Male	2,940	84.5	481	77.6
Region				
Missing	1	0.0	0	0.0
Asia	1,593	45.8	286	46.1
ROW	1,884	54.2	334	53.9
ECOG performance status				
0	2,109	60.6	347	56.0
1+	1,369	39.4	273	44.0
Child–Pugh score				
Missing	4	0.1	3	0.5
A	3435	98.8	601	96.9
B	39	1.1	16	2.6
Aetiology of chronic liver disease				
Missing	203	5.8	36	5.8
Alcohol	205	5.9	36	5.8
HBV	1,383	39.8	235	37.9
HCV	723	20.8	148	23.9
Mixed	166	4.8	29	4.7
Other	700	20.1	119	19.2
Unknown	98	2.8	17	2.7
Prior systemic therapy				
Yes	2,158	62.0	385	62.1
No	1,320	38.0	235	37.9
Macrovascular invasion				
Missing	2	0.1	1	0.2
Absence	2,495	71.7	431	69.5
Presence	981	28.2	188	30.3
Extrahepatic spread				
Absence	1,127	32.4	178	28.7
Presence	2,351	67.6	442	71.3
Type of treatment				
ICI monotherapy	225	6.5	33	5.3
ICI combination	482	13.9	102	16.5
TKI monotherapy	1,656	47.6	312	50.3
VEGF inhibitors	407	11.7	73	11.8
Placebo	708	20.4	100	16.1

ECOG, European Collaborative Oncology Group; ICI, immune checkpoint inhibitor; ROW, rest of the world; TKI, tyrosine kinase inhibitor; VEGF, vascular endothelial growth factor.

Baseline characteristics

Baseline characteristics of the whole population are summarised in Table 1 following stratification by ATB exposure. In the overall population, 83% (n = 3,421) of patients were male, 46% (n = 1,879) were enrolled in Asia, and 54% (n = 2,218) were from the rest of the world. Leading aetiologic factors for chronic liver disease included 39% (n = 1,618) hepatitis B infection, 21% (n = 871) hepatitis C infection, 5% (n = 195) mixed infection, 6% (n = 241) alcohol use, 20% (n = 819) other causes, 2.8% (n = 115) unknown aetiologic factors, and 6% (n = 239) missing.

At baseline, Child–Pugh class was A in 4,036 patients (98%), and most trial participants carried a diagnosis of HCC with evidence of extrahepatic spread (n = 2,793, 68%), whereas macrovascular invasion was present in 1,169 patients (29%). At baseline, 2,543 patients (62%) had received at least one line of systemic anticancer therapy.

ATB exposure is associated with worse outcome in patients who receive systemic therapy in HCC

In total, 4,036 patients (98%) were eligible for analysis of ATB exposure in relation to OS and PFS outcomes, after exclusion of 62 patients with Child–Pugh class B (n = 55) and seven patients with missing Child–Pugh class information. Patients with Child–Pugh class B disease were not examined owing to their small sample size. In all treatment modalities (ICIs, TKIs, and placebo),

Table 2. The relationship between antibiotic use and efficacy outcomes in patients with unresectable/advanced HCC.

	Unadjusted analyses		Adjusted using IPTW*	
	No antibiotic use	Antibiotic use	No antibiotic use	Antibiotic use
**All patients**				
Sample size				
No. of patients	3,435	601	3,171	555
Effect size	3,435	601	3,725.77	3,730.41
PFS				
Median (95% CI), months	4.2 (4.1, 4.5)	3.6 (3.0, 4.0)	4.2 (4.0, 4.4)	3.6 (2.7, 4.0)
HR† (95% CI)	1.36 (1.23, 1.50)		1.29 (1.22,1.36)	
OS				
Median (95% CI), months	10.8 (10.4, 11.3)	8.6 (7.9, 9.1)	10.6 (10.3, 11.1)	8.7 (7.8, 9.6)
HR† (95% CI)	1.42 (1.29, 1.57)		1.36 (1.29,1.43)	
**Tyrosine kinase inhibitor group**				
Sample size				
No. of patients	1,640	303	1,469	278
Effect size	1,640	303	1,746.74	1,752.47
PFS				
Median (95% CI), months	5.6 (5.5, 5.7)	3.7 (3.6, 4.3)	5.5 (5.4,5.6)	3.9 (3.6,4.9)
HR (95% CI)	1.40 (1.22, 1.61)		1.29 (1.19,1.39)	
OS				
Median (95% CI), months	12.2 (11.6, 13.1)	8.4 (7.1, 9.8)	11.9 (11.1,12.5)	8.8 (7.4,10.4)
HR (95% CI)	1.51 (1.31, 1.73)		1.40 (1.30,1.52)	
**Immunotherapy group**				
Sample size				
No. of patients	696	129	660	120
Effect size	696	129	779.56	788.67
PFS				
Median (95% CI), months	8.2 (7.6, 8.4)	8.3 (6.8, 8.9)	8.3 (7.6, 8.5)	6.8 (5.4, 8.3)
HR† (95% CI)	1.24 (0.97, 1.59)		1.52 (1.34, 1.73)	
OS				
Median (95% CI), months	11.4 (10.6, 12.1)	10.7 (9.1, 11.8)	11.5 (10.7,12.3)	10.7 (9.0,12.1)
HR† (95% CI)	1.24 (0.98,1.56)		1.22 (1.08,1.38)	
**Placebo group**				
Sample size				
No. of patients	697	97	675	92
Effect size	697	97	766.69	772.45
PFS				
Median (95% CI), months	1.87 (1.81, 1.93)	1.61 (1.41, 1.84)	1.9 (1.8,1.9)	1.6 (1.4,1.9)
HR (95% CI)	1.29 (1.03, 1.62)		1.23 (1.11,1.37)	
OS				
Median (95% CI), months	8.2 (7.4, 8.9)	4.4 (3.7, 6.6)	8.1 (7.3,8.9)	5.2 (3.7,7.7)
HR (95% CI)	1.58 (1.25, 1.99)		1.40 (1.25,1.57)	

ECOG, European Collaborative Oncology Group; HR, hazard ratio; IPTW, inverse probability of treatment weighting; OS, overall survival; PFS, progression-free survival; VEGF, vascular endothelial growth factor.

* The weights were derived from a propensity score (IPTW) model that included the following baseline variables as covariates: age, race, sex, region, ECOG performance status, aetiology of chronic liver disease, presence of macrovascular invasion, presence of extrahepatic disease, and receipt of prior lines of treatment.

† HR is stratified by treatment group (tyrosine kinase inhibitor or immune checkpoint inhibitor alone or immune checkpoint inhibitor combination or placebo or VEGF for all patients, or immune checkpoint inhibitor alone or immune checkpoint inhibitor combination for immune checkpoint inhibitor group).

unadjusted and adjusted (by IPTW) PFS and OS analyses were conducted to compare patients by ATB exposure groups.

Baseline characteristics in the various groups were found to be generally well balanced between the ATB-exposed and ATB-unexposed groups before and after weighting (Tables S2–S4). Exceptions are among TKI recipients where ATB was more frequently administered to female patients than to male patients (21 *vs.* 15%), and to patients who received prior systemic therapy for HCC (58 *vs.* 51%), and were less frequently administered to patients who had extrahepatic disease (65 *vs.* 71%). All variables were well balanced after weighting (Table S2).

Before weighting, in the overall patient population, ATB-exposed patients (n = 601) experienced worse PFS (median, 3.6 months in ATB-exposed patients *vs.* 4.2 months; HR 1.36; 95% CI 1.23, 1.50) and worse OS (median, 8.6 months in ATB-exposed patients *vs.* 10.8 months; HR 1.42; 95% CI 1.29, 1.57) than did their ATB-unexposed counterparts (n = 3,435). After IPTW, the survival estimates in ATB-exposed patients (n = 555) *vs.* ATB-unexposed patients (n = 3,171) were comparable with the unadjusted results for both PFS (median, 3.6 months in ATB-exposed patients *vs.* 4.2 months; HR 1.29; 95% CI 1.22, 1.36) and OS (median, 8.7 *vs.* 10.6 months; HR 1.36; 95% CI 1.23, 1.43).

ATB exposure is associated with worse efficacy and survival across various therapeutic modalities

To determine whether the effect of ATB exposure on PFS and OS seen in the overall patient population could be separately

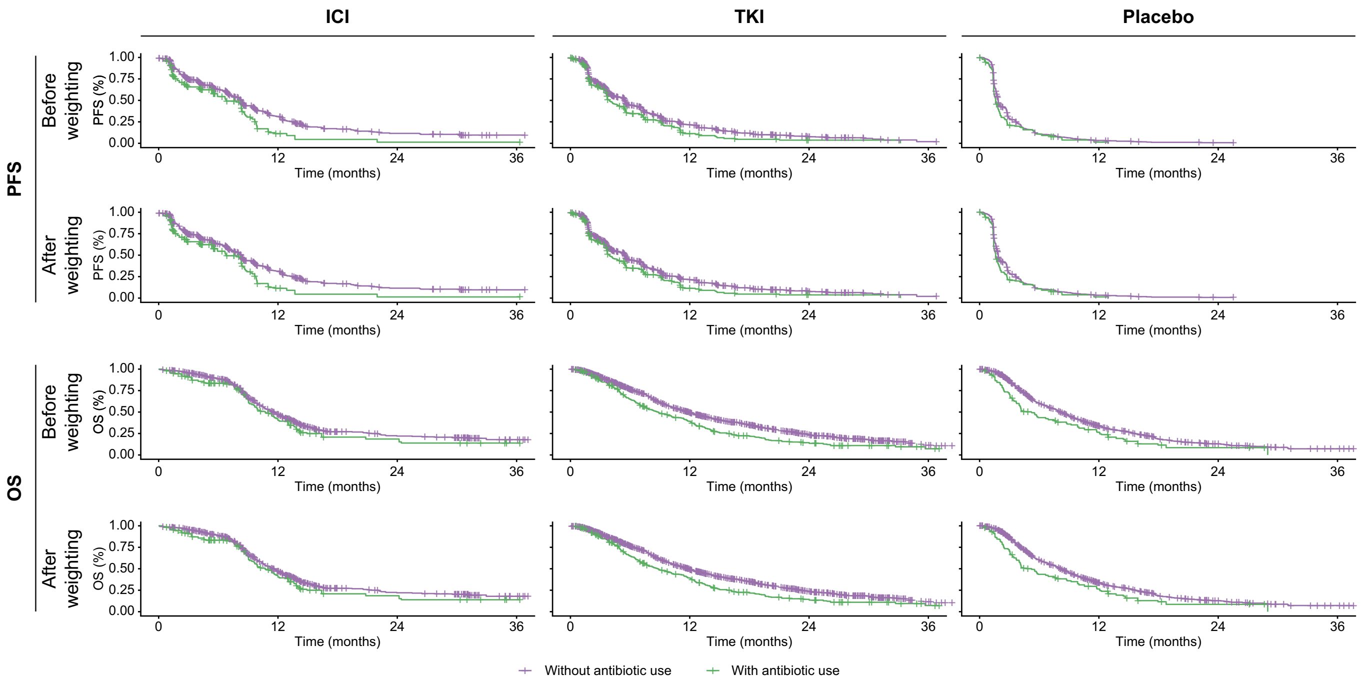

Fig. 1. Kaplan–Meier curves illustrating estimates for PFS and OS stratified according to antibiotic exposure before and after propensity score weighting in patients who received ICIs, TKIs, and placebo. ICI, immune checkpoint inhibitor; OS, overall survival; PFS, progression-free survival; TKI, tyrosine kinase inhibitor.

reproduced in patients treated with ICI, TKI, and placebo, we performed unmatched estimates for PFS in ATB-exposed *vs.* unexposed patients.

As shown in Table 2, worse PFS and OS in patients treated with TKIs and placebo was observed, whereas for the ICI group, median PFS (median, 8.3 months in ATB-exposed patients *vs.* 8.2 months; HR 1.24; 95% CI 0.97, 1.59) and OS (median, 10.7 months in ATB-exposed patients *vs.* 11.4 months; HR 1.24; 95% CI 0.98, 1.56) was not significantly different.

After adjusting for baseline differences using IPTW, estimates for PFS and OS showed a consistent worsening in ATB-exposed patients *vs.* ATB-unexposed counterparts. Fig. 1 illustrates Kaplan–Meier estimates of PFS and OS by ATB exposure groups across TKI-treated, ICI-treated, and placebo-treated cohorts.

Next, we evaluated ICI combination regimens separately from ICI monotherapy.

As shown in Table S5, the unadjusted analyses indicate an association between ATB exposure and worse PFS from ICI monotherapy regimens (median, 2.1 months in ATB-exposed patients *vs.* 4.0 months; HR 1.51; 95% CI 1.00, 2.28). Following IPTW, we found evidence of worse PFS and OS outcomes in ICI monotherapy and combination therapy for patients with ATB exposure (Fig. S1).

Comparable results were also observed after propensity score matching (Tables S6–S10).

Discussion

The liver immune microenvironment is naturally geared towards spontaneous immune suppression. As chronic liver disease worsens over time, increased intestinal permeability, bacterial overgrowth, or impaired clearance of microbial metabolites by Kupffer cells may increase the translocation of gut microbial species, leading to unopposed pro-inflammatory signalling within the liver and systemically.[32,33] An altered intestinal homoeostasis not only is pathogenic in HCC but is intimately linked to the natural progression and prognosis of liver cancer.[34] Although evidence gathered in other tumours has revealed an intimate relationship between diversity and taxonomic features of the gut microbiome and responsiveness to ICI, data on HCC are still preliminary.[35]

Similarly, the effect of ATB, a broad class of therapies associated with the highest disruption of and potential to induce long-lasting changes to the gut microbiome,[36] has not been univocally confirmed to affect responsiveness and survival of patients with HCC treated with ICI.[28,29]

In this study, the first to evaluate the relationship between ATB exposure and outcomes in a large series of patients prospectively recruited to landmark clinical trials of unresectable/advanced HCC, we provide data that ATB exposure within 30 days of treatment initiation is associated with worse PFS and OS across patients treated with a wide variety of systemic therapies for HCC including TKI and ICI monotherapy and combinations.

In ICI recipients, the negative association between ATB and survival outcomes was preserved across ICI monotherapy and combinations including dual PD-1/VEGF pathway blockade, the novel standard of care in advanced HCC.[10]

In patients receiving immunotherapy, enrichment of certain stool bacterial species including *Akkermansia*, *Ruminococcus*, and *Bifidobacteria* is associated with a higher likelihood of response from ICI.[19,37,38] The selective pressure of broad-spectrum ATB exposure on the gut microbiome leads to the expansion of *Bacteroides* and commensal *Clostridia*,[39] which can in turn facilitate recruitment and activation of immunosuppressive cells including T-regulatory and myeloid-derived suppressor cells – all negatively associated with ICI response.[40] One recent study suggested faecal microbial transplantation to restore sensitivity to PD-1

inhibition in patients with advanced melanoma,[41] potentially highlighting the central role of the gut microbiome in driving adaptive resistance to ICI.

Although the relationship between ATB and outcomes is supported by mechanistic studies and analyses of clinical data in ICI recipients, the finding of a detrimental effect across multiple treatment arms including placebo in our study is in contrast with evolving evidence in oncological indications other than HCC, where the detrimental effect of ATB pretreatment on response and survival is restricted to ICI therapy and not to chemotherapy[25] or targeted therapies unless prescribed after immune-modulating therapies.[42] Previously published retrospective evidence in sorafenib recipients had suggested that receipt of broad-spectrum ATB therapy before sorafenib was associated with worse survival in patients with advanced HCC,[43] concordantly with our study data.

Reasons and mechanisms explaining the shorter survival observed in patients treated with ATB and TKI are unknown. A significant component of the efficacy of sorafenib potentially resides in the capacity to improve the T-effector/T-regulatory cell ratio and augment the proportion of interferon-γ-secreting $CD8^+$ T cells.[44] Similarly, other molecularly targeted therapies such as lenvatinib[45] and cabozantinib[46] exert their pleiotropic antitumour effects by acting on lymphoid and myeloid constituents of the tumour microenvironment both alone and in synergy with concurrent PD-1 pathway inhibition. In addition, evidence from renal cell carcinoma suggests that VEGF pathway inhibitors can directly alter intestinal homoeostasis,[18] highlighting how ATB may plausibly alter a complex bidirectional relationship between TKI therapy and survival by acting synergistically on composition and function of the gut microbiome.

In attempting to interpret potentially causal as opposed to purely associative links between ATB and outcome, a clinically important finding from our study is the demonstration of a detrimental effect of ATB on survival observed in patients who received placebo. Although it is possible that the postulated changes in the gut microbiome and immune function mediated by ATB preconditioning could have exerted their influence even in the absence of anticancer therapy, an alternative explanation that we should consider is whether ATB dosing before treatment might select for patients with uncontrolled infection, who are at higher risk for recurrent severe infections, or who have rapidly progressive disease before treatment allocation. Patients with cirrhosis carry a higher risk of bacterial infections compared with the general population,[47] and both empirical and prophylactic ATB therapy is indicated in response to decompensation of chronic liver disease to improve survival outcomes.[48] In the absence of adequately powered translational studies evaluating the effect of ATB therapy on stool samples and peripheral immune cells, support for a causative or an associative relationship between ATB and outcome remains speculative.

Although no direct causative role between ATB and outcome can be inferred from our retrospective analysis, the utilisation of prospectively accrued, geographically heterogeneous patient cohorts, carefully selected on the basis of stringent inclusion criteria and with careful documentation of response and survival outcomes lends significant credibility to the associations observed. In addition, use of methods such as IPTW that adjust for key clinicopathologic features of cirrhosis and HCC affords sufficient robustness to our survival estimates across ATB strata and reduces the risk of bias. Among important limitations to our study are the issues associated with retrospectively examining data from completed studies. These included missing information on the number and sizes of HCC lesions, baseline AFP levels, and BCLC stages in a proportion of patients, which did not allow us to consider these variables for matching. Moreover, the restriction of ATB exposure window to 30 days might have failed to capture clinically meaningful exposures that extended beyond 30 days from treatment initiation as well as the impact of ATB treatment later during the course of systemic therapy.[20] In addition, the lack of information on indication (*i.e.* prophylactic *vs.* therapeutic), ATB class, and course length made further subgroup analyses impossible and might represent a limitation. Furthermore, we acknowledge significant heterogeneity in the studies selected for this analysis: the entirety of TKI studies included phase III trials powered on OS, whereas a number of ICI studies included phase I/II studies powered on safety outcomes and response rates, a finding that may have affected the maturity of OS and PFS data pooled for our analysis. The line of therapy was also heterogeneous across studies, and none of our OS estimates could be adjusted for post-study therapy. Finally, causes of death are not available, preventing further analyses on infection-related outcomes.

In conclusion, our study demonstrates that ATB exposure may be associated with worse PFS and OS in patients receiving ICI monotherapy as well as combinations. Although the positive association identified in our study does not prove causality, the hypothesis that an ATB-mediated gut dysbiosis may correlate with reduced antitumour efficacy is provocative and resonates with evolving clinical and translational knowledge in the field. The association between ATB exposure and outcomes from TKI therapy and placebo highlights the uniquely complex interplay between cirrhosis, cancer, risk of infection, and the pleiotropic effect of molecular therapies for HCC. To overcome the complexity of these distinctively intertwined relationships, prospective studies should investigate the role of gut microbial diversity as a determinant of outcome in patients with HCC undergoing systemic therapy. The rapid expansion of immunotherapy combinations, for which no predictive biomarker of benefit exists, highlights the importance of this stream of research as a pathway towards personalised medicine in HCC.[10]

Abbreviations

ATB, antibiotics; CTLA-4, cytotoxic T-cell lymphocyte associated antigen-4; ECOG, European Collaborative Oncology Group; HCC, hepatocellular carcinoma; HR, hazard ratio; ICI, immune checkpoint inhibitor; IPTW, inverse probability of treatment weighting; MSD, mean squared difference; OS, overall survival; PD-1, programmed cell death-1; PD-L1, programmed cell death ligand-1; PFS, progression-free survival; RECIST, Response Evaluation Criteria in Solid Tumours; TKI, tyrosine kinase inhibitor; VEGF, vascular endothelial growth factor.

Financial support

AD is supported by the NIHR Imperial BRC and by grant funding from EASL (Andrew Burroughs Fellowship) and Cancer Research UK (RCCPDB-Nov21/100008). DJP is supported by grant funding from the Wellcome Trust Strategic Fund (PS3416) and the Associazione Italiana per la Ricerca sul

Cancro (AIRC MFAG 25697), and acknowledges grant support from the Cancer Treatment and Research Trust (CTRT) and infrastructural support by the Imperial Experimental Cancer Medicine Centre and the NIHR Imperial Biomedical Research Centre. AC is supported by the NIHR Imperial BRC.

Conflicts of interest

DJP received lecture fees from ViiV Healthcare, Bayer Healthcare, EISAI, BMS, and Roche; travel expenses from BMS and Bayer Healthcare; and consulting fees for Mina Therapeutics, DaVolterra, Mursla, IPSEN, Exact Sciences, Avamune, EISAI, Roche, and Astra Zeneca. DJP received research funding (to institution) from MSD, GSK, and BMS. BS received travel support from AbbVie, Gilead, and Ipsen. AC received consulting fees from MSD, Astra Zeneca, Roche, and BMS. He also received speaker fees from Novartis, Astra Zeneca, and EISAI. AD received educational grant support for conference attendance by Roche. There are no other personal or financial conflicts of interest to disclose.

Please refer to the accompanying ICMJE disclosure forms for further details.

Authors' contributions

Study concept and design: DJP, LP, JS, PMK. Acquisition of data: XL, PMK, GW, JS. Analysis and interpretation of data: XL, PMK, DJP, LP, GW, AD, CAMF, BS, DRR, RP, MT, SC, SL, LF, AC. Drafting of the manuscript: DJP. Critical revision of the manuscript for important intellectual content: all authors. Statistical analysis: XL, PMK. Obtained funding: DJP. Study supervision: DJP, LP.

Data availability statement

The data that support the findings of this study are available from the US FDA. Restrictions apply to the availability of these data, and employees of the FDA performed all raw data analyses.

Supplementary data

Supplementary data to this article can be found online at https://doi.org/10.1016/j.jhepr.2023.100747.

References

Author names in bold designate shared co-first authorship

[1] Fulgenzi CAM, D'Alessio A, Talbot T, Gennari A, Openshaw MR, Demirtas CO, et al. New frontiers in the medical therapy of hepatocellular carcinoma. Chemotherapy 2022;67:164–172.

[2] Finn RS, Merle P, Granito A, Huang YH, Bodoky G, Pracht M, et al. Outcomes of sequential treatment with sorafenib followed by regorafenib for HCC: additional analyses from the phase III RESORCE trial. J Hepatol 2018;69:353–358.

[3] Haber PK, Puigvehí M, Castet F, Lourdusamy V, Montal R, Tabrizian P, et al. Evidence-based management of hepatocellular carcinoma: systematic review and meta-analysis of randomized controlled trials (2002–2020). Gastroenterology 2021;161:879–898.

[4] Galluzzi L, Chan TA, Kroemer G, Wolchok JD, López-Soto A. The hallmarks of successful anticancer immunotherapy. Sci Transl Med 2018;10:eaat7807.

[5] Llovet JM, Castet F, Heikenwalder M, Maini MK, Mazzaferro V, Pinato DJ, et al. Immunotherapies for hepatocellular carcinoma. Nat Rev Clin Oncol 2022;19:151–172.

[6] Yau T, Park JW, Finn RS, Cheng AL, Mathurin P, Edeline J, et al. Nivolumab versus sorafenib in advanced hepatocellular carcinoma (CheckMate 459): a randomised, multicentre, open-label, phase 3 trial. Lancet Oncol 2022;23:77–90.

[7] Zhu AX, Finn RS, Edeline J, Cattan S, Ogasawara S, Palmer D, et al. Pembrolizumab in patients with advanced hepatocellular carcinoma previously treated with sorafenib (KEYNOTE-224): a non-randomised, open-label phase 2 trial. Lancet Oncol 2018;19:940–952.

[8] Shek D, Read SA, Nagrial A, Carlino MS, Gao B, George J, et al. Immune-checkpoint inhibitors for advanced hepatocellular carcinoma: a synopsis of response rates. Oncologist 2021;26:e1216–e1225.

[9] Finn RS, Qin S, Ikeda M, Galle PR, Ducreux M, Kim TY, et al. Atezolizumab plus bevacizumab in unresectable hepatocellular carcinoma. N Engl J Med 2020;382:1894–1905.

[10] Muhammed A, D'Alessio A, Enica A, Talbot T, Fulgenzi CAM, Nteliopoulos G, et al. Predictive biomarkers of response to immune checkpoint inhibitors in hepatocellular carcinoma. Expert Rev Mol Diagn 2022;22:253–264.

[11] Cortellini A, Bersanelli M, Buti S, Cannita K, Santini D, Perrone F, et al. A multicenter study of body mass index in cancer patients treated with anti-PD-1/PD-L1 immune checkpoint inhibitors: when overweight becomes favorable. J Immunother Cancer 2019;7:57.

[12] **Muhammed A**, **Fulgenzi CAM**, **Dharmapuri S**, Pinter M, Balcar L, Scheiner B, et al. The systemic inflammatory response identifies patients with adverse clinical outcome from immunotherapy in hepatocellular carcinoma. Cancers 2021;14:186.

[13] Scheiner B, Pomej K, Kirstein MM, Hucke F, Finkelmeier F, Waidmann O, et al. Prognosis of patients with hepatocellular carcinoma treated with immunotherapy – development and validation of the CRAFITY score. J Hepatol 2022;76:353–363.

[14] Pinato DJ, Marron TU, Mishra-Kalyani PS, Gong Y, Wei G, Szafron D, et al. Treatment-related toxicity and improved outcome from immunotherapy in hepatocellular cancer: evidence from an FDA pooled analysis of landmark clinical trials with validation from routine practice. Eur J Cancer 2021;157:140–152.

[15] Hussain N, Naeem M, Pinato DJ. Concomitant medications and immune checkpoint inhibitor therapy for cancer: causation or association? Hum Vaccin Immunother 2021;17:55–61.

[16] Cortellini A, Tucci M, Adamo V, Stucci LS, Russo A, Tanda ET, et al. Integrated analysis of concomitant medications and oncological outcomes from PD-1/PD-L1 checkpoint inhibitors in clinical practice. J Immunother Cancer 2020;8:e001361.

[17] Pinato DJ, Gramenitskaya D, Altmann DM, Boyton RJ, Mullish BH, Marchesi JR, et al. Antibiotic therapy and outcome from immune-checkpoint inhibitors. J Immunother Cancer 2019;7:287.

[18] Derosa L, Routy B, Fidelle M, Iebba V, Alla L, Pasolli E, et al. Gut bacteria composition drives primary resistance to cancer immunotherapy in renal cell carcinoma patients. Eur Urol 2020;78:195–206.

[19] **Derosa L**, **Routy B**, **Thomas AM**, Iebba V, Zalcman G, Friard S, et al. Intestinal Akkermansia muciniphila predicts clinical response to PD-1 blockade in patients with advanced non-small-cell lung cancer. Nat Med 2022;28:315–324.

[20] Derosa L, Hellmann MD, Spaziano M, Halpenny D, Fidelle M, Rizvi H, et al. Negative association of antibiotics on clinical activity of immune checkpoint inhibitors in patients with advanced renal cell and non-small-cell lung cancer. Ann Oncol 2018;29:1437–1444.

[21] Pinato DJ, Howlett S, Ottaviani D, Urus H, Patel A, Mineo T, et al. Association of prior antibiotic treatment with survival and response to immune checkpoint inhibitor therapy in patients with cancer. JAMA Oncol 2019;5:1774–1778.

[22] Weinstock C, Maher VE, Fernandes LL, Tang S, Agrawal S, Brave MH, et al. Impact of antibiotic use on clinical outcomes in patients with urothelial cancer receiving a programmed death protein 1 or programmed death ligand 1 (anti-PD-1/L1) antibody. J Clin Oncol 2019;37:4557. https://doi.org/10.1200/JCO.2019.37.15_suppl.4557.

[23] Weinstock C, Bandaru P, Fernandes LL, Chang E, Girvin A, Tang S, et al. Impact of timing of antibiotic use on clinical outcomes in patients with urothelial cancer treated with immune checkpoint inhibitors (ICIs). J Clin Oncol 2020;38(15_suppl). 5045–5045.

[24] Maher VE, Fernandes LL, Weinstock C, Tang S, Agarwal S, Brave M, et al. Analysis of the association between adverse events and outcome in patients receiving a programmed death protein 1 or programmed death ligand 1 antibody. J Clin Oncol 2019;37:2730–2737.

[25] Cortellini A, Di Maio M, Nigro O, Leonetti A, Cortinovis DL, Aerts JG, et al. Differential influence of antibiotic therapy and other medications on oncological outcomes of patients with non-small cell lung cancer treated with first-line pembrolizumab versus cytotoxic chemotherapy. J Immunother Cancer 2021;9:e002421.

[26] Cortellini A, Ricciuti B, Facchinetti F, Alessi JVM, Venkatraman D, Dall'Olio FG, et al. Antibiotic-exposed patients with non-small-cell lung cancer preserve efficacy outcomes following first-line chemo-immunotherapy. Ann Oncol 2021;32:1391–1399.

[27] Elkrief A, Derosa L, Kroemer G, Zitvogel L, Routy B. The negative impact of antibiotics on outcomes in cancer patients treated with immunotherapy: a new independent prognostic factor? Ann Oncol 2019;30:1572–1579.

[28] Cheung KS, Lam LK, Seto WK, Leung WK. Use of antibiotics during immune checkpoint inhibitor treatment is associated with lower survival in hepatocellular carcinoma. Liver Cancer 2021;10:606–614.

[29] Fessas P, Naeem M, Pinter M, Marron TU, Szafron D, Balcar L, et al. Early antibiotic exposure is not detrimental to therapeutic effect

from immunotherapy in hepatocellular carcinoma. Liver Cancer 2021;10:583–592.

[30] D'Alessio A, Fulgenzi CAM, Nishida N, Schönlein M, von Felden J, Schulze K, et al. Preliminary evidence of safety and tolerability of atezolizumab plus bevacizumab in patients with hepatocellular carcinoma and Child–Pugh A and B cirrhosis: a real-world study. Hepatology 2022;76:1000–1012.

[31] Pinato DJ, Kaneko T, Saeed A, Pressiani T, Kaseb A, Wang Y, et al. Immunotherapy in hepatocellular cancer patients with mild to severe liver dysfunction: adjunctive role of the ALBI grade. Cancers 2020;12:1862.

[32] Tao X, Wang N, Qin W. Gut microbiota and hepatocellular carcinoma. Gastrointest Tumors 2015;2:33–40.

[33] Pinato DJ, Guerra N, Fessas P, Murphy R, Mineo T, Mauri FA, et al. Immune-based therapies for hepatocellular carcinoma. Oncogene 2020;39:3620–3637.

[34] Schwabe RF, Greten TF. Gut microbiome in HCC – mechanisms, diagnosis and therapy. J Hepatol 2020;72:230–238.

[35] Zheng Y, Wang T, Tu X, Huang Y, Zhang H, Tan D, et al. Gut microbiome affects the response to anti-PD-1 immunotherapy in patients with hepatocellular carcinoma. J Immunother Cancer 2019;7:193.

[36] Pérez-Cobas AE, Gosalbes MJ, Friedrichs A, Knecht H, Artacho A, Eismann K, et al. Gut microbiota disturbance during antibiotic therapy: a multi-omic approach. Gut 2013;62:1591–1601.

[37] **Gopalakrishnan V**, **Spencer C**, Nezi L, Reuben A, Andrews M, Karpinets T, et al. Gut microbiome modulates response to anti–PD-1 immunotherapy in melanoma patients. Science 2018;359:97–103.

[38] Matson V, Fessler J, Bao R, Chongsuwat T, Zha Y, Alegre ML, et al. The commensal microbiome is associated with anti-PD-1 efficacy in metastatic melanoma patients. Science 2018;359:104–108.

[39] **Spakowicz D**, **Hoyd R**, Muniak M, Husain M, Bassett JS, Wang L, et al. Inferring the role of the microbiome on survival in patients treated with immune checkpoint inhibitors: causal modeling, timing, and classes of concomitant medications. BMC Cancer 2020;20:383.

[40] Naqash AR, Kihn-Alarcón AJ, Stavraka C, Kerrigan K, Maleki Vareki S, Pinato DJ, et al. The role of gut microbiome in modulating response to immune checkpoint inhibitor therapy in cancer. Ann Transl Med 2021;9:1034.

[41] Baruch EN, Youngster I, Ben-Betzalel G, Ortenberg R, Lahat A, Katz L, et al. Fecal microbiota transplant promotes response in immunotherapy-refractory melanoma patients. Science 2021;371:602–609.

[42] Lalani AA, Xie W, Braun DA, Kaymakcalan M, Bosse D, Steinharter JA, et al. Effect of antibiotic use on outcomes with systemic therapies in metastatic renal cell carcinoma. Eur Urol Oncol 2020;3:372–381.

[43] **Pomej K**, **Balcar L**, Scheiner B, Semmler G, Meischl T, Mandorfer M, et al. Antibiotic therapy is associated with worse outcome in patients with hepatocellular carcinoma treated with sorafenib. J Hepatocell Carcinoma 2021;8:1485–1493.

[44] Kalathil SG, Hutson A, Barbi J, Iyer R, Thanavala Y. Augmentation of IFN-γ^+ $CD8^+$ T cell responses correlates with survival of HCC patients on sorafenib therapy. JCI Insight 2019;4:e130116.

[45] Torrens L, Montironi C, Puigvehí M, Mesropian A, Leslie J, Haber PK, et al. Immunomodulatory effects of lenvatinib plus anti-programmed cell death protein 1 in mice and rationale for patient enrichment in hepatocellular carcinoma. Hepatology 2021;74:2652–2669.

[46] **Esteban-Fabró R**, **Willoughby CE**, Piqué-Gili M, Montironi C, Abril-Fornaguera J, Peix J, et al. Cabozantinib enhances anti-PD1 activity and elicits a neutrophil-based immune response in hepatocellular carcinoma. Clin Cancer Res 2022;28:2449–2460.

[47] Fernández J, Navasa M, Gómez J, Colmenero J, Vila J, Arroyo V, et al. Bacterial infections in cirrhosis: epidemiological changes with invasive procedures and norfloxacin prophylaxis. Hepatology 2002;35:140–148.

[48] **Fernández J**, **Tandon P**, Mensa J, Garcia-Tsao G. Antibiotic prophylaxis in cirrhosis: Good and bad. Hepatology 2016;63:2019–2031.

Journal of Hepatology, Volume *5*

Supplemental information

Association between antibiotics and adverse oncological outcomes in patients receiving targeted or immune-based therapy for hepatocellular carcinoma

David J. Pinato, Xiaoxue Li, Pallavi Mishra-Kalyani, Antonio D'Alessio, Claudia A.M. Fulgenzi, Bernhard Scheiner, Matthias Pinter, Guo Wei, Julie Schneider, Donna R. Rivera, Richard Pazdur, Marc R. Theoret, Sandra Casak, Steven Lemery, Lola Fashoyin-Aje, Alessio Cortellini, and Lorraine Pelosof

Association between antibiotics and adverse oncological outcomes in patients receiving targeted or immune-based therapy for hepatocellular carcinoma

David J. Pinato, Xiaoxue Li, Pallavi Mishra-Kalyani, Antonio D'Alessio, Claudia A. M. Fulgenzi, Bernhard Scheiner, Matthias Pinter, Guo Wei, Julie Schneider, Donna R. Rivera, Richard Pazdur, Marc R. Theoret, Sandra Casak, Steven Lemery, 'Lola Fashoyin-Aje, Alessio Cortellini, Lorraine Pelosof

Table of contents

FIGURES

Fig. S1. **Page 3**
Kaplan-Meier curves depicting the effect of antibiotic use on progression-free survival (Panels A, B) and overall survival (Panels C, D) before (A, C) and after (B, D) propensity score weighting.

TABLES

Table S1 **Page 6**
Use of antibiotics within 30 days of initiation of study treatment

Table S2 **Page 7**
Baseline patient characteristics stratified by antibiotic exposure prior to and after inverse probability of treatment weighting (IPTW): Tyrosine kinase inhibitor group.

Table S3 **Page 8**
Baseline patient characteristics stratified by antibiotic exposure prior to and after inverse probability of treatment weighting (IPTW): immunotherapy group.

Table S4 **Page 9**
Baseline patient characteristics stratified by antibiotic exposure prior to and after inverse probability of treatment weighting (IPTW): placebo group.

Table S5 **Page 10**
The relationship between antibiotic use and efficacy outcomes in patients with unresectable/advanced HCC treated with immune checkpoint inhibitor (ICI) combinations or monotherapy.

Table S6 **Page 11**
Baseline patient characteristics stratified by antibiotic exposure prior to and after propensity score matching: Tyrosine kinase inhibitor group.

Table S7 **Page 12**
Baseline patient characteristics stratified by antibiotic exposure prior to and after propensity score matching: immunotherapy group.

Table S8 **Page 13**
Baseline patient characteristics stratified by antibiotic exposure prior to and after propensity score matching: placebo group.

Table S9 **Page 14**
The relationship between antibiotic use and efficacy outcomes in patients with unresectable/advanced HCC before and after propensity score matching.

Table S10 **Page 15**
The relationship between antibiotic use and efficacy outcomes in patients with unresectable/advanced HCC treated with immune checkpoint inhibitor (ICI) combinations or monotherapy before and after propensity score matching.

Figures

Fig. S1. Kaplan-Meier curves depicting the effect of antibiotic use on PFS (Panels A, B) and OS (Panels C, D) before (A, C) and after (B, D) IPTW.

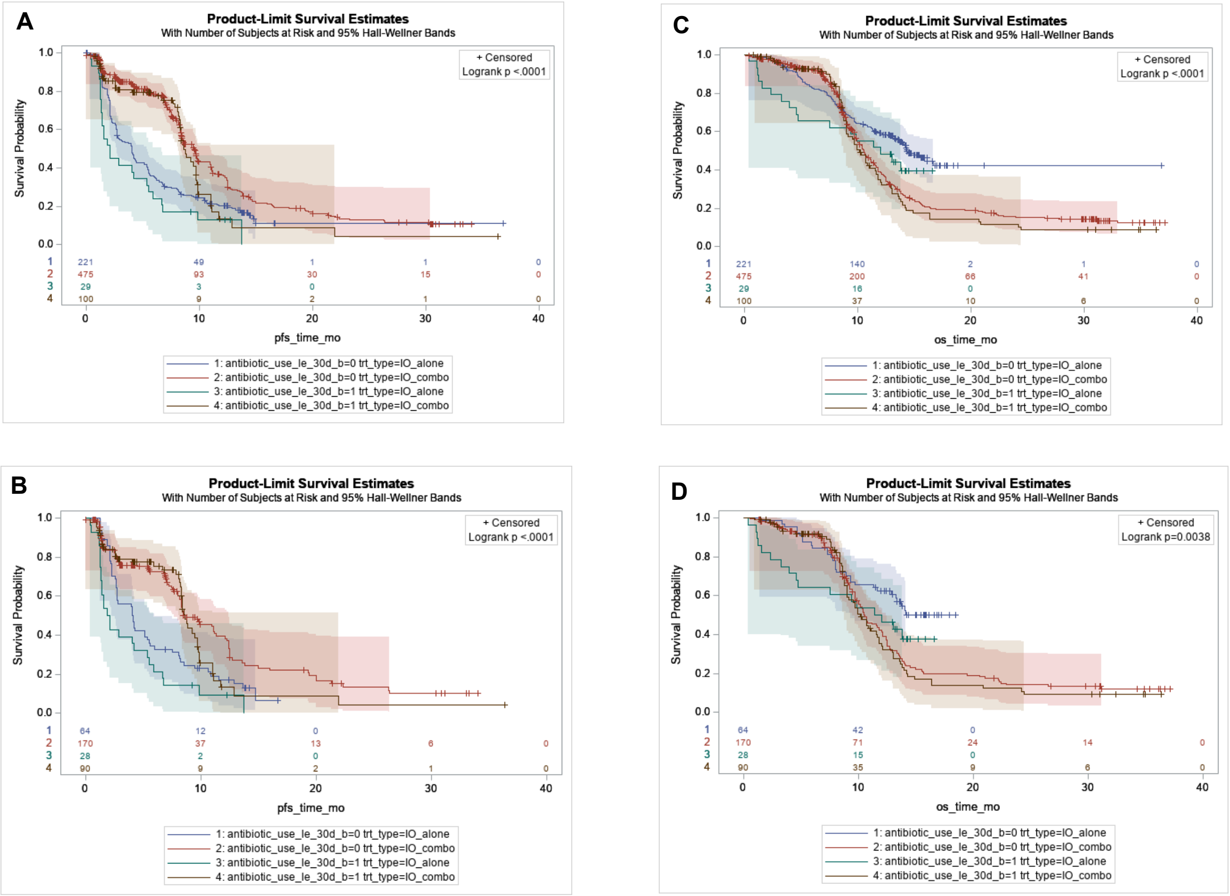

TABLES

Table S1. Use of antibiotics within 30 days of initiation of study treatment.

Antibiotic Use	Placebo (n = 808)	Tyrosine kinase inhibitor (n = 1968)	Vascular Endothelial Growth Factor (VEGF) inhibitor (n = 480)	Immunotherapy (n = 842)	All patients (n = 4098)
None or not within 30 days	708 (88%)	1656 (84%)	407 (85%)	707 (84%)	3478 (85%)
Used within 30 days	100 (12%)	312 (16%)	73 (15%)	135 (16%)	620 (15%)
**Antibiotic Duration**					
≤10 days of exposure	49 (49%)	140 (45%)	41 (56%)	51 (38%)	281 (45%)
>10 days of exposure	51 (51%)	172 (55%)	32 (44%)	84 (62%)	339 (55%)

Table S2. Baseline patient characteristics stratified by antibiotic exposure prior to and after inverse probability of treatment weighting (IPTW): TKI group.

	No antibiotic use N = 1640 Effect size = 1640		Antibiotic use N = 303 Effect size = 303		Mean Squared Differences before Propensity Score Weighting	No antibiotic use N = 1469 Effect size = 1746.74		Antibiotic use N = 278 Effect size = 1752.47		Mean Squared Differences before Propensity Score Weighting
**Variable**	**Mean/N**	**STD/%**	**Mean/N**	**STD/%**		**Mean/N**	**STD/%**	**Mean/N**	**STD/%**	
**Age**										
Mean/SD	61.96	11.67	62.38	11.70	0.035	61.87	12.53	61.91	29.09	0.002
Median/Range	63	19-88	63	27-88		63	19-88	62	27-88	
**Race**										
Asian	899	54.8	166	54.8	-0.001	977	55.9	975	55.6	-0.006
Other/Not reported	131	8.0	33	10.9	0.10	162	9.3	156	8.9	-0.01
White	610	37.2	104	34.3	-0.06	607	34.8	622	35.5	0.01
**Sex**										
Female	247	15.1	64	21.1	0.16	274	15.7	273	15.6	-0.003
Male	1393	84.9	239	78.9	-0.16	1473	84.3	1480	84.4	0.003
**Region**										
Asia	836	51.0	153	50.5	-0.01	903	51.7	905	51.7	-0.001
Rest of world	804	49.0	150	49.5	0.01	844	48.3	847	48.3	0.001
**ECOG PS**										
0	1010	61.6	178	58.7	-0.06	1079	61.8	1110	63.3	0.03
1	630	38.4	125	41.3	0.06	667	38.2	642	36.7	-0.03
**Etiology**										
Non-viral	353	21.5	68	22.4	0.02	420	24.0	427	24.3	0.01
Viral	1118	68.2	210	69.3	0.02	1327	76.0	1326	75.7	-0.01
**Prior Systemic Therapy**										
Yes	957	58.4	154	50.8	-0.15	917	52.5	924	52.7	0.004
No	683	41.7	149	49.2	0.15	830	47.5	828	47.3	-0.004
**Macrovascular**										
Absence	1224	74.6	218	71.9	-0.06	1289	73.8	1298	74.1	0.006
Presence	414	25.2	85	28.1	0.06	458	26.2	455	25.9	-0.006
**Extrahepatic**										
Absence	572	34.9	88	29.0	-0.13	597	34.2	606	34.6	0.01
Presence	1068	65.1	215	71.0	0.13	1150	65.8	1147	65.4	-0.01

Table S3. Baseline patient characteristics stratified by antibiotic exposure prior to and after inverse probability of treatment weighting (IPTW): immunotherapy group.

	**No antibiotic use** N = 696 Effect size = 696		**Antibiotic use** N = 129 Effect size = 129		**Mean Squared Differences before Propensity Score Weighting**	**No antibiotic use** N = 660 Effect size = 779.56		**Antibiotic use** N = 120 Effect size = 788.67		**Mean Squared Differences before Propensity Score Weighting**
**Variable**	**Mean/N**	**STD/%**	**Mean/N**	**STD/%**		**Mean/N**	**STD/%**	**Mean/N**	**STD/%**	
**Age**										
Mean/SD	62.75	11.51	63.39	11.80	0.05	63.49	11.70	62.99	11.76	-0.04
Median/Range	64	18-88	64	34-88		64	18-88	64	34-88	
**Race**										
Asian	337	48.4	65	50.4	0.04	397	50.9	408	51.8	0.02
Other/Not reported	35	5.0	11	8.5	0.14	45	5.7	42	5.3	-0.02
White	312	44.8	51	39.5	-0.11	338	43.3	338	42.9	-0.01
**Sex**										
Female	129	18.5	28	21.7	0.08	145	18.5	148	18.8	0.005
Male	567	81.5	101	78.3	-0.08	635	81.5	641	81.2	-0.005
**Region**										
Asia	307	44.1	53	41.1	-0.06	356	45.7	368	46.6	0.02
Rest of world	388	55.7	76	58.9	0.06	423	54.3	421	53.4	-0.02
**ECOG PS**										
0	434	62.4	72	55.8	-0.13	473	60.6	472	59.9	-0.015
1	262	37.6	57	44.2	0.13	307	39.4	316	40.1	0.015
**Etiology**										
Non-viral	222	31.9	49	38.0	0.13	255	32.7	252	32.0	-0.01
Viral	458	65.8	74	57.4	-0.17	525	67.3	537	68.0	0.01
**Prior Systemic Therapy**										
No	355	51.0	76	58.9	0.16	395	50.7	390	49.4	-0.03
Yes	341	49.0	53	41.1	-0.16	384	49.3	399	50.6	0.03
**Immunotherapy**										
Monotherapy	221	31.8	29	22.5	-0.21	240	30.9	263	33.3	0.05
Combination	475	68.2	100	77.5	0.21	539	69.1	526	66.7	-0.05
**Macrovascular**										
Absence	478	68.7	90	69.8	0.02	533	68.3	552	69.9	0.03
Presence	218	31.3	38	29.5	-0.04	247	31.7	237	30.1	-0.03
**Extrahepatic**										
Absence	236	33.9	38	29.5	-0.10	249	31.9	276	35.0	0.07
Presence	460	66.1	91	70.5	0.10	531	68.1	513	65.0	-0.07

Table S4. Baseline patient characteristics stratified by antibiotic exposure prior to and after inverse probability of treatment weighting (IPTW): placebo group.

Variable	**No antibiotic use** N = 697 Effect size = 697		**Antibiotic use** N = 97 Effect size = 97		**Mean Squared Differences before Propensity Score Weighting**	**No antibiotic use** N = 675 Effect size = 766.69		**Antibiotic use** N = 92 Effect size = 722.45		**Mean Squared Differences before Propensity Score Weighting**
	**Mean/N**	**STD/%**	**Mean/N**	**STD/%**		**Mean/N**	**STD/%**	**Mean/N**	**STD/%**	
**Age**										
Mean/STD	62.50	11.09	62.55	12.11	0.004	62.26	11.61	62.01	11.62	-0.02
Median/Range	63	23-86	63	24-85		63	23-86	63	24-85	
**Race**										
Asian	300	43.0	38	39.2	-0.08	335	43.7	339	43.9	0.005
Other/Not reported	69	9.9	9	9.3	-0.02	78	10.2	85	11.0	0.03
White	315	45.2	46	47.4	0.04	354	46.1	348	45.1	-0.02
**Sex**										
Female	89	12.8	24	24.7	0.31	106	13.8	104	13.4	-0.01
Male	608	87.2	73	75.3	-0.31	661	86.2	669	86.6	0.01
**Region**										
Asia	432	62.0	61	62.9	-0.02	298	38.9	309	40.0	0.02
Rest of world	418	60.0	45	46.4	0.02	469	61.1	463	60.0	-0.02
**ECOG PS**										
0	279	40.0	52	53.6	-0.27	452	59.0	458	59.2	0.01
1	10	1.4	1	1.0	0.27	314	41.0	315	40.8	-0.01
**Etiology**										
Non-viral	264	37.9	30	30.9	-0.15	286	37.3	304	39.3	0.04
Viral	423	60.7	66	68.0	0.15	480	62.7	469	60.7	-0.04
**Prior Systemic Therapy**										
No	0	-	0	-	-	0	-	0	-	-
Yes	697	-	97	-	-	766.69	-	772.45	-	-
**Macrovascular**										
Absence	489	70.2	62	63.9	-0.13	536	69.9	550	71.3	0.03
Presence	208	29.8	35	36.1	0.13	231	30.1	222	28.7	-0.03
**Extrahepatic**										
Absence	197	28.3	26	26.8	-0.03	214	27.9	208	26.9	-0.02
Presence	500	71.7	71	73.2	0.03	553	72.1	565	73.1	0.02

Table S5. The relationship between antibiotic use and efficacy outcomes in patients with unresectable/advanced HCC treated with immune checkpoint inhibitor (ICI) combinations or monotherapy.

	**Unadjusted Analyses**		**Adjusted using IPTW ***	
	**No Antibiotic Use**	**Antibiotic Use**	**No Antibiotic Use**	**Antibiotic Use**
**Immune checkpoint inhibitor combinations**				
Sample size				
# patients	475	100	448	92
Effect size	475	100	539.76	542.65
**Progression-free survival (PFS)**				
Median (95% CI), mo	9.6 (8.5, 9.8)	8.6 (8.3, 9.7)	9.7 (8.5, 11.0)	8.6 (8.3, 9.7)
HR** (95% CI)	1.33 (0.97, 1.81)		1.50 (1.26, 1.77)	
**Overall survival (OS)**				
Median (95% CI), mo	10.5 (10.0, 11.2)	10.2 (9.0, 11.6)	10.6 (10.2, 11.4)	9.9 (9.0, 11.6)
HR** (95% CI)	1.13 (0.87, 1.47)		1.14 (0.99, 1.32)	
**Immune checkpoint inhibitor monotherapy**				
Sample size				
# patients	221	29	212	28
Effect Size	221	29	240.20	228.58
**Progression-free survival (PFS)**				
Median (95% CI), mo	4.0 (2.8, 4.4)	2.1 (1.3, 5.4)	4.0 (2.8, 4.4)	2.1 (1.3, 5.4)
HR (95% CI)	1.51 (1.00, 2.28)		1.49 (1.22 ,1.80)	
**Overall survival (OS)**				
Median (95% CI), mo	14.3 (13.2, NE)	12.0 (4.6, NE)	14.4 (13.2, NE)	13.0 (4.6, NE)
HR (95% CI)	1.47 (0.88, 2.45)		1.31 (1.02, 1.68)	

** The weights were derived from a propensity score (IPTW) models which included the following baseline variables as covariates: age, race, gender, region, European Collaborative Oncology Group (ECOG) performance status, etiology of chronic liver disease, presence of macrovascular invasion, presence of extrahepatic disease and receipt of prior lines of treatment.*
*** HR is stratified by immune checkpoint inhibitor alone or immune checkpoint inhibitor combination.*

Table S6. Baseline patient characteristics stratified by antibiotic exposure prior to and after propensity score matching: Tyrosine kinase inhibitor (TKI) group.

Variable	No antibiotic use N = 1640		Antibiotic use N = 303		Mean Squared Differences before matching	No antibiotic use N = 548		Antibiotic use N = 276		Mean Squared Differences after matching
	Mean/N	STD/%	Mean/N	STD/%		Mean/N	STD/%	Mean/N	STD/%	
**Age**	61.96	11.67	62.38	11.70	0.035	62.26	11.61	62.01	11.62	-0.021
**Race**										
Asian	899	54.8	166	54.8	-0.001	296	54.0	149	54.0	-0.001
Other/Not reported	131	8.0	33	10.9	0.10	64	11.7	33	12.0	0.01
White	610	37.2	104	34.3	-0.06	188	34.3	94	34.1	-0.01
**Sex**										
Female	247	15.1	64	21.1	0.16	107	19.5	57	20.7	0.03
Male	1393	84.9	239	78.9	-0.16	441	80.5	219	79.3	-0.03
**Region**										
Asia	836	51.0	153	50.5	-0.01	271	49.5	138	50.0	0.01
Rest of world	804	49.0	150	49.5	0.01	277	50.5	138	50.0	-0.01
**ECOG**										
0	1010	61.6	178	58.7	-0.06	325	59.3	161	58.3	-0.02
1	630	38.4	125	41.3	0.06	223	40.7	115	41.7	0.02
**Etiology**										
Non-viral	353	21.5	68	22.4	0.02	135	24.6	68	24.6	0.00
Viral	1118	68.2	210	69.3	0.02	413	75.4	208	75.4	0.00
**Prior Therapy**										
Yes	957	58.4	154	50.8	-0.15	265	48.4	129	46.7	-0.03
No	683	41.7	149	49.2	0.15	283	51.6	147	53.3	0.03
**Macrovascular**										
Absence	1224	74.6	218	71.9	-0.06	396	72.3	200	72.5	0.004
Presence	414	25.2	85	28.1	0.06	152	27.7	76	27.5	-0.004
**Extrahepatic**										
Absence	572	34.9	88	29.0	-0.13	149	27.2	79	28.6	0.03
Presence	1068	65.1	215	71.0	0.13	399	72.8	197	71.4	-0.03

Table S7. Baseline patient characteristics stratified by antibiotic exposure prior to and after propensity score matching: immunotherapy group.

Variable	No antibiotic use N = 696		Antibiotic use N = 129		Mean Squared Differences before matching	No antibiotic use N = 234		Antibiotic use N = 118		Mean Squared Differences after matching
	Mean/N	STD/%	Mean/N	STD/%		Mean/N	STD/%	Mean/N	STD/%	
**Age**	62.75	11.51	63.39	11.80	0.05	63.49	11.70	62.99	11.76	-0.04
**Race**										
Asian	337	48.4	65	50.4	0.04	115	49.1	62	52.5	0.07
Other/Not reported	35	5.0	11	8.5	0.14	21	9.0	10	8.5	-0.02
White	312	44.8	51	39.5	-0.11	98	41.9	46	39.0	-0.06
**Sex**										
Female	129	18.5	28	21.7	0.08	47	20.1	24	20.3	0.006
Male	567	81.5	101	78.3	-0.08	187	79.9	94	79.7	-0.006
**Region**										
Asia	307	44.1	53	41.1	-0.06	95	40.6	51	43.2	0.05
Rest of world	388	55.7	76	58.9	0.06	139	59.4	67	56.8	-0.05
**ECOG**										
0	434	62.4	72	55.8	-0.13	133	56.8	67	56.8	-0.001
1	262	37.6	57	44.2	0.13	101	43.2	51	43.2	0.001
**Etiology**										
Non-viral	222	31.9	49	38.0	0.13	96	41.0	46	39.0	-0.04
Viral	458	65.8	74	57.4	-0.17	138	59.0	72	61.0	0.04
**Prior Therapy**										
No	355	51.0	76	58.9	0.16	122	52.1	66	55.9	0.08
Yes	341	49.0	53	41.1	-0.16	112	47.9	52	44.1	-0.08
**Immunotherapy**										
Monotherapy	221	31.8	29	22.5	-0.21	64	27.4	28	23.7	-0.08
Combination	475	68.2	100	77.5	0.21	170	72.6	90	76.3	0.08
**Macrovascular**										
Absence	478	68.7	90	69.8	0.02	158	67.5	81	68.6	0.02
Presence	218	31.3	38	29.5	-0.04	76	32.5	37	31.4	-0.02
**Extrahepatic**										
Absence	236	33.9	38	29.5	-0.10	64	27.4	34	28.8	0.03
Presence	460	66.1	91	70.5	0.10	170	72.6	84	71.2	-0.03

Table S8. Baseline patient characteristics stratified by antibiotic exposure prior to and after propensity score matching: placebo group.

Variable	No antibiotic use N = 697		Antibiotic use N = 97		Mean Squared Differences before matching	No antibiotic use N = 175		Antibiotic use N = 91		Mean Squared Differences after matching
	Mean/N	STD/%	Mean/N	STD/%		Mean/N	STD/%	Mean/N	STD/%	
**Age**	62.50	11.09	62.55	12.11	0.004	62.77	10.72	62.56	12.14	-0.018
**Race**										
Asian	300	43.0	38	39.2	-0.08	69	39.4	37	40.7	0.025
Other/Not reported	69	9.9	9	9.3	-0.02	21	12.0	9	9.9	-0.07
White	315	45.2	46	47.4	0.04	85	48.6	45	49.5	0.02
**Sex**										
Female	89	12.8	24	24.7	0.31	41	23.4	23	25.3	0.04
Male	608	87.2	73	75.3	-0.31	134	76.6	68	74.7	-0.04
**Region**										
Asia	432	62.0	61	62.9	-0.02	63	36.0	35	38.5	0.05
Rest of world	418	60.0	45	46.4	0.02	112	64.0	56	61.5	-0.05
**ECOG**										
0	279	40.0	52	53.6	-0.27	90	51.4	44	48.4	-0.06
1	10	1.4	1	1.0	0.27	85	48.6	47	51.6	0.06
**Etiology**										
Non-viral	264	37.9	30	30.9	-0.15	50	28.6	28	30.8	0.05
Viral	423	60.7	66	68.0	0.15	125	71.4	63	69.2	-0.05
**Prior Therapy**										
No	0	-	0	-	-	0	-	0	-	-
Yes	697	-	97	-	-	175	-	91	-	-
**Macrovascular**										
Absence	489	70.2	62	63.9	-0.13	122	69.7	59	64.8	-0.10
Presence	208	29.8	35	36.1	0.13	53	30.3	32	35.2	0.10
**Extrahepatic**										
Absence	197	28.3	26	26.8	-0.03	54	30.9	23	25.3	-0.12
Presence	500	71.7	71	73.2	0.03	121	69.1	68	74.7	0.12

Table 9. The relationship between antibiotic use and efficacy outcomes in patients with unresectable/advanced HCC before and after propensity score matching.

	**Unadjusted Analyses**		**Adjusted using propensity score**	
	**No Antibiotic Use**	**Antibiotic Use**	**No Antibiotic Use**	**Antibiotic Use**
**All patients**				
Sample size				
# patients	3435	601	1091	550
**Progression-free survival (PFS)**				
Median (95% CI), months	4.2 (4.1, 4.5)	3.6 (3.0, 4.0)	5.4 (4.3, 5.5)	3.6 (3.0, 4.0)
HR* (95% CI)	1.36 (1.23, 1.50)		1.39 (1.23 ,1.57)	
**Overall survival (OS)**				
Median (95% CI), months	10.8 (10.4, 11.3)	8.6 (7.9, 9.1)	11.1 (10.4, 11.8)	8.6 (7.8, 9.2)
HR* (95% CI)	1.42 (1.29, 1.57)		1.41 (1.25 ,1.59)	
**Tyrosine kinase inhibitor Group**				
Sample size				
# patients	1640	303	548	276
**Progression-free survival (PFS)**				
Median (95% CI), months	5.6 (5.5, 5.7)	3.7 (3.6, 4.3)	5.5 (4.7 ,5.6)	3.7 (3.6 ,4.3)
HR (95% CI)	1.40 (1.22, 1.61)		1.27 (1.08 ,1.51)	
**Overall survival (OS)**				
Median (95% CI), months	12.2 (11.6, 13.1)	8.4 (7.1, 9.8)	12.1 (10.7 ,13.9)	8.6 (7.1 ,10.1)
HR (95% CI)	1.51 (1.31, 1.73)		1.41 (1.19 ,1.68)	
**Immunotherapy Group**				
Sample size				
# patients	696	129	234	118
**Progression-free survival (PFS)**				
Median (95% CI), months	8.2 (7.6, 8.4)	8.3 (6.8, 8.9)	8.1 (7.0, 8.5)	8.3 (6.7, 8.6)
HR* (95% CI)	1.39 (1.08, 1.78)		1.41 (1.06, 1.71)	
**Overall survival (OS)**				
Median (95% CI), months	11.4 (10.6, 12.1)	10.7 (9.1, 11.8)	11.8 (10.4 ,12.7)	10.7 (9.1 ,11.8)
HR* (95% CI)	1.19 (0.94 ,1.50)		1.19 (0.91 ,1.57)	
**Placebo Group**				
Sample size				
# patients	697	97	175	91
**Progression-free survival (PFS)**				
Median (95% CI), months	1.87 (1.81, 1.93)	1.61 (1.41, 1.84)	1.8 (1.7 ,2.1)	1.61 (1.41 ,1.91)
HR (95% CI)	1.29 (1.03, 1.62)		1.31 (1.00 ,1.71)	
**Overall survival (OS)**				
Median (95% CI), months	8.2 (7.4, 8.9)	4.4 (3.7, 6.6)	6.87 (5.45 ,9.40)	4.17 (3.45 ,5.88)
HR (95% CI)	1.58 (1.25, 1.99)		1.54 (1.16 ,2.05)	

** HR is stratified by treatment group (tyrosine kinase inhibitor or immune checkpoint inhibitor alone or immune checkpoint inhibitor combination or Placebo or VEGF for all patients, immune checkpoint inhibitor alone or immune checkpoint inhibitor combination for immune checkpoint inhibitor group).*

Table S10. The relationship between antibiotic use and efficacy outcomes in patients with unresectable/advanced HCC treated with immune checkpoint inhibitor (ICI) combinations or monotherapy before and after propensity score matching.

	**Unadjusted Analyses**		**Adjusted using propensity score**	
	**No Antibiotic Use**	**Antibiotic Use**	**No Antibiotic Use**	**Antibiotic Use**
**Immune checkpoint inhibitor combinations**				
Sample size				
# patients	475	100	177	90
**Progression-free survival (PFS)**				
Median (95% CI), months	9.6 (8.5, 9.8)	8.6 (8.3, 9.7)	11.0 (8.4, 12.1)	8.6 (8.3, 9.7)
HR (95% CI)	1.33 (0.97, 1.81)		1.61 (1.12, 2.33)	
**Overall survival (OS)**				
Median (95% CI), months	10.5 (10.0, 11.2)	10.2 (9.0, 11.6)	11.4 (10.4, 12.1)	10.2 (9.0, 11.6)
HR (95% CI)	1.13 (0.87, 1.47)		1.23 (0.91, 1.68)	
**Immune checkpoint inhibitor monotherapy**				
Sample size				
# patients	221	29	56	28
**Progression-free survival (PFS)**				
Median (95% CI), months	4.0 (2.8, 4.4)	2.1 (1.3, 5.4)	4.0 (2.6, 5.4)	2.1 (1.3, 4.2)
HR (95% CI)	1.51 (1.00, 2.28)		1.48 (0.92, 2.38)	
**Overall survival (OS)**				
Median (95% CI), months	14.3 (13.2, NE)	12.0 (4.6, NE)	14.4 (10.5, NE)	11.9 (4.6, NE)
HR (95% CI)	1.47 (0.88, 2.45)		1.61 (0.87, 2.97)	

NE = not estimable
